# Intermolecular biparatopic trapping of ErbB2 prevents compensatory activation of PI3K/AKT via RAS–p110 crosstalk

**DOI:** 10.1038/ncomms11672

**Published:** 2016-06-03

**Authors:** Rastislav Tamaskovic, Martin Schwill, Gabriela Nagy-Davidescu, Christian Jost, Dagmar C. Schaefer, Wouter P. R. Verdurmen, Jonas V. Schaefer, Annemarie Honegger, Andreas Plückthun

**Affiliations:** 1 Department of Biochemistry, University of Zurich, Winterthurerstr. 190, Zurich 8057, Switzerland; 2 Institute of Laboratory Animal Science, University of Zurich, Winterthurerstr. 190, Zurich 8057, Switzerland

## Abstract

Compensatory mechanisms, such as relief of AKT-ErbB3-negative feedback, are known to desensitize ErbB2-dependent tumours to targeted therapy. Here we describe an adaptation mechanism leading to reactivation of the PI3K/AKT pathway during trastuzumab treatment, which occurs independently of ErbB3 re-phosphorylation. This signalling bypass of phospho-ErbB3 operates in ErbB2-overexpressing cells via RAS-PI3K crosstalk and is attributable to active ErbB2 homodimers. As demonstrated by dual blockade of ErbB2/RAS and ErbB3 by means of pharmacological inhibition, RNA interference or by specific protein binders obstructing the RAS–p110α interaction, both routes must be blocked to prevent reactivation of the PI3K/AKT pathway. Applying these general principles, we developed biparatopic designed ankyrin repeat proteins (DARPins) trapping ErbB2 in a dimerization-incompetent state, which entail pan-ErbB inhibition and a permanent OFF state in the oncogenic signalling, thereby triggering extensive apoptosis in ErbB2-addicted tumours. Thus, these novel insights into mechanisms underlying network robustness provide a guide for overcoming adaptation response to ErbB2/ErbB3-targeted therapy.

## Abstract (web summary)

Targeted therapy of ErbB2-dependent tumours often provokes an adaptive response leading to reactivation of the PI3K/AKT pathway. Here the authors identify an ErbB3-independent compensatory mechanism comprising Ras/PI3K activation directly by ErbB2, and develop biparatopic panErbB inhibitors to block this mode of resistance.

## Graphical abstract

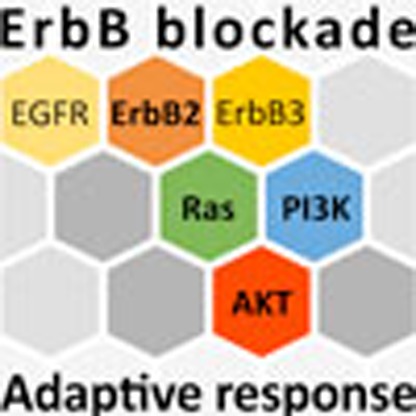

Human epidermal growth factor receptor type 2 (ErbB2/HER2/neu) is an orphan receptor adopting a constitutively extended conformation, which serves as preferred heterodimerization partner for the ligand-activated receptors of the ErbB family. The physiological function of this non-autonomous co-receptor is thus to act primarily as an amplifier of ErbB signalling^1^. Nevertheless, under the conditions of pathological overexpression of ErbB2, as it occurs in >20% of invasive breast cancer and, to a lesser extent, in gastric or ovarian cancers^2^, ErbB2 drives tumorigenesis by spontaneously forming signalling-competent homodimers, ligand-independent heterodimers with ErbB3 as well as larger receptor clusters^3^,^4^,^5^,^6^.

As for many other signalling, genetic or metabolic networks, the ErbB network displays two steady states, that is, bistability, with ligands transiently switching it from the OFF to the ON state^1^. Importantly, the pathological *ERBB2* amplification maintains ErbB signalling constitutively active, thereby fixing the ON state. On the other hand, scale-free networks frequently evolve reliance on few highly connected nodes, entailing increased system fragility, as blockade of these central hubs may cause the entire network to fail. Since malignant diseases efficiently hijack key regulatory elements of the host networks, these essential hubs can consequently become their ‘Achilles heels'^7^. Such a vulnerability of cancer cells, also referred to as ‘oncogene addiction', may thus provide valuable opportunity for targeted therapy.

In fact, knockdown of *ERBB2* expression triggers apoptosis in ErbB2-addicted breast cancer cells, while barely having an effect on cells that do not overexpress this gene^8^. Analogously, blockade of the canonical PI3K/AKT/mTOR pathway by small-molecule inhibitors elicits apoptosis in ErbB2-overexpressing tumour cells that become addicted also to PI3K signalling, as it conveys proliferation and survival signals downstream of ErbB2/3 receptors. On the other hand, activating *PIK3CA* mutations make the tumours refractory to ErbB2-targeted therapy, and the response may be restored by co-inhibition of PI3K. Thus, the possibility to induce tumour cell death by incapacitating critical nodes of the oncogenic network provides a strong rationale for targeting the ErbB2/3 oncogenic unit and the downstream signalling in ErbB2-addicted tumours.

Nevertheless, therapeutic monoclonal antibodies (mAbs) specific for ErbB2 target only few epitopes and show rather poor clinical efficacy in overall long-term survival even as part of combination therapy regimens. The humanized mAb trastuzumab is directed against the membrane-proximal domain IV of ErbB2 (ref. 9). It specifically inhibits the growth of breast cancer cell lines addicted to ErbB2, inducing cell cycle arrest in the G1 phase. Although it was thought earlier that trastuzumab inhibits ErbB2 signalling, more recent studies have shown that trastuzumab does not decrease ErbB2 phosphorylation^10^. Instead, other conceivable mechanisms, such as dissociation of the ligand-independent ErbB2–ErbB3 heterodimers and consequently dephosphorylation of ErbB3 (ref. 3), are a likely component of the enigmatic mode of action of trastuzumab in ErbB2-overexpressing tumours. Another approved ErbB2-binding antibody, pertuzumab, binds adjacent to the domain II dimerization arm, thereby disturbing the heterodimerization of ErbB2 with the other ligand-bound epidermal growth factor receptor (EGFR) family members^11^. Pertuzumab thus abrogates solely the ligand-stimulated growth without affecting the proliferation of tumour cells driven by ErbB2 overexpression^3^. Similarly, the antibodies neutralizing or locking ErbB3 in a tethered conformation have failed to raise a cytotoxic response in ErbB2-overexpressing tumours, even though ErbB3 is thought to be indispensable for ErbB2 oncogenic signalling^12^,^13^,^14^.

Thus, none of the therapeutic ErbB2/3-targeting mAbs can fully exploit the addiction to ErbB2 as a tumour's fragile point for therapeutic intervention. Apart from the moderate activity, multiple mechanisms of innate or acquired resistance have also been described that often neutralize the anti-tumour effect of trastuzumab^15^. Acquired resistance arises due to permanent alterations such as PI3K-activating mutations^16^, PTEN loss^17^, expression of truncated ErbB2 (ref. 15), or long-term adaptive changes comprising upregulated expression of ErbB family receptors, cognate RTKs, the respective ligands^10^, or activation of co-operating signaling proteins such as Src and CDCP1 (refs 18, 19).

A series of tyrosine kinase inhibitors (TKIs) blocking the ErbB intracellular kinase domains have also been developed, comprising both reversible and irreversible inhibitors with specificity ranging from mono- or dual-specificity EGFR and ErbB2 inhibitors to broad-spectrum inhibitors. TKIs elicit stronger and faster biological responses *in vitro* than the highly specific yet less potent ErbB2-targeting antibodies^5^. However, these responses tend to be short-lived because of rapid emergence of adaptive resistance caused by the reactivation of ErbB3 signalling^20^,^21^ and, consequently, the rebound of AKT activity due to relief of a negative feedback loop with the downstream effector FoxO3a^22^. Such phenomena resulting from the robustness of ErbB oncogenic signalling also translate to low clinical efficacy, as was illustrated by rather sobering data from clinical trials, demonstrating limited activity of lapatinib either as single agent or in combination with trastuzumab^23^,^24^,^25^.

Therefore, other approaches comprising inhibitors of PI3K/AKT/mTOR pathway, as support of ErbB2 blockade, are currently being explored, showing encouraging results in preclinical models and ongoing clinical trials^26^. These approaches, based on understanding of the compensatory mechanisms, are aimed at preventing the emergence of resistance in tumours, which show an initial response to blockade of ErbB2. Elucidation of the feedbacks and crosstalks within signalling pathways underlying the adaptive response thus hold promise to assist the development of new rational combination strategies with improved short-term clinical efficacy^27^ as well as long-term prevention of development of acquired resistance.

In the present study, we describe yet another important adaptation mechanism operating in ErbB2-dependent tumour cells, which causes the reactivation of the PI3K/AKT pathway even in the presence of de-phosphorylated ErbB3 receptor. This mechanism of PI3K/AKT activation bypasses ErbB3 via crosstalk with the ErbB2-bound small-GTPase RAS, explaining the rapid desensitization to trastuzumab even in the absence of pathway-activating mutations. Although some compensatory mechanisms involving elevated expression of potent activators of RAS such as EGFR, IGF-1R or c-Met have been described^15^, thus anticipating a non-oncogenic function of RAS in mediating resistance to ErbB2 blockade, the mechanistic explanation is still overdue. Our findings provide for the first time a direct signalling evidence linking RAS to evasion of apoptosis in ErbB2-dependent tumours, particularly under the conditions where ErbB2 does not become efficiently inhibited.

Applying these new insights, we improved upon the traditional concepts of ErbB2 targeting by designing and mechanistically dissecting new biparatopic agents that trap ErbB2 in a dimerization-incompetent state thereby entailing the pan-ErbB inhibition in ErbB2-addicted tumours. This targeting approach was shown to disarm all compensatory mechanisms, resulting in apoptosis induction by enforcing the permanent OFF state in the ErbB2-driven oncogenic network.

## Results

We constructed biparatopic proteins which specifically bind to the ectodomain of ErbB2 (ErbB2_ECD). They are composed of two designed ankyrin repeat proteins^28^ (DAPRins). The biparatopic DARPins with highest anti-tumour activity, referred to as 6L1G and 9L1H (Supplementary Experimental Procedures), were fusions of a subdomain I-binding DARPin^29^ followed by a subdomain IV-binding DARPin^30^, which are connected through a short (Gly_4_Ser) linker (called ‘L1'). Crystal structures of the DARPins in complex with their target ErbB2 subdomain^31^ allowed detailed structural modelling, and together with binding analyses, revealed that both binding units are intermolecularly engaged in binding to two ErbB2 receptor molecules on the cell surface. Such a crosslinking of ErbB2 receptors by biparatopic DARPins was inferred to induce trapping of ErbB2 (ref. 31), enforcing a geometry of the ErbB2_ECD that is incompatible with receptor dimerization or alternative associations, such as tetramers or higher oligomers^6^, as well as with trans-activation of receptor kinase domains^32^,^33^. By now dissecting the mechanism of action of these potent biparatopic agents as compared with ErbB2-targeting mAbs, we could elucidate compensatory responses upon blockade of ErbB2/ErbB3, which mediate adaptive resistance in ErbB2-overexpressing cancers towards mAb treatment.

### Apoptosis requires dephosphorylation of both ErbB2 and ErbB3

**Figure 1:**
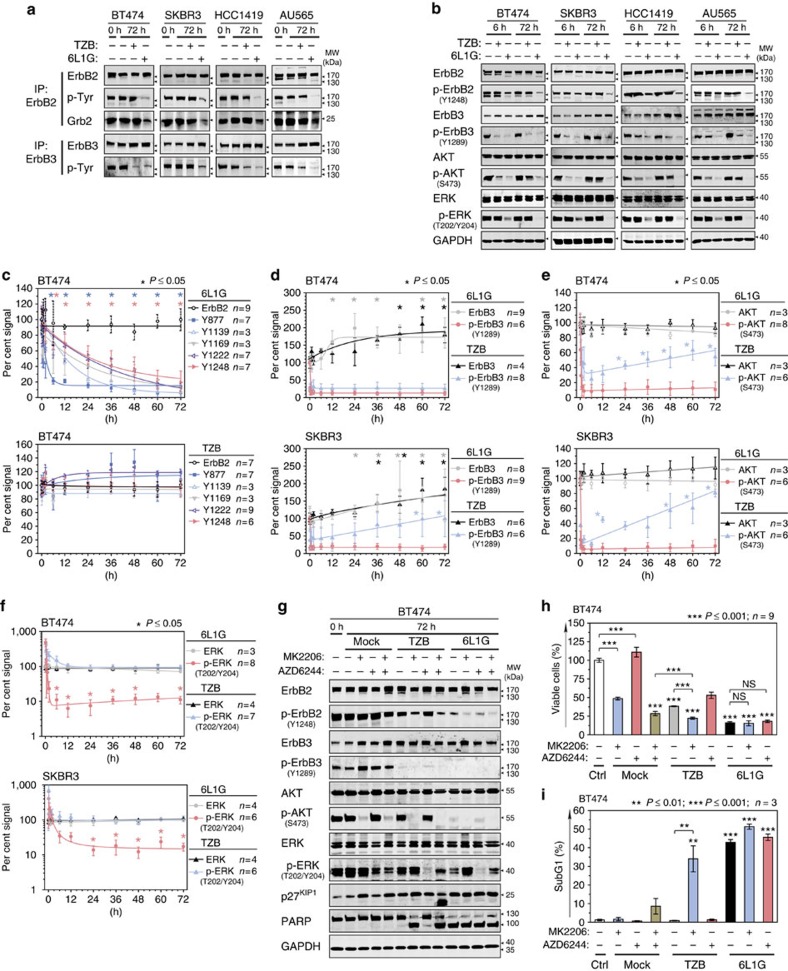
Dynamic signalling responses to ErbB2 blockade. (**a**) Biparatopic anti-ErbB2 DARPin (6L1G) inhibits phosphorylation of both ErbB2 and ErbB3 while trastuzumab (TZB) attenuates only p-ErbB3. ErbB2-overexpressing cell lines were treated with 100 nM of 6L1G or TZB; ErbB2 or ErbB3 was immunoprecipitated and total phosphotyrosine content (p-Tyr) was determined. Co-IP analysis of Grb2 binding to ErbB2 is also shown. (**b**) Sustained inhibition of both AKT and ERK signalling pathways after 6L1G treatment versus transient decrease of p-AKT after 6 h of TZB treatment and consecutive p-AKT rebound after 72 h. Cancer cell lines were treated as above and whole-cell extracts were analysed. (**c**–**f**) Dynamic signalling events induced by TZB and 6L1G: quantitative western blot time course analysis. Data are means±s.d. of integrated intensities (I.I., *cf.* Li-COR Odyssey manual), with 100% referring to I.I. of untreated cells (0 h). (**c**) Expression of ErbB2 and reduced phosphorylation at specific Tyr-residues (color-coded (Y877 to Y1248), *P value versus level at 0 h), (**d**) expression of ErbB3 and level of p-ErbB3 after 6L1G or TZB treatment (colour-coded significance levels; ErbB3 expression upregulation, *P value versus 0 h; p-ErbB3 Y1289 re-phosphorylation, *P value versus 30 min TZB) (**e**) expression of AKT and level of p-AKT (p-AKT re-phosphorylation, *P value versus 6 h TZB) (**f**) expression of ERK1/2 and level of p-ERK1/2 in BT474 and SKBR3 cells (p-ERK dephosphorylation, *P value versus 0 h). For representative blots see Supplementary Fig. 1A,B. (All statistical analyses are one-way ANOVA on ranks and *post hoc* Dunnett's test.) (**g**–**i**) Inhibition of downstream signalling (**g**) and ensuing effects of proliferation (XTT assay; **h**) or induction of apoptosis (FACS; **i**) after 72 h of treatment with 1 μM of AKT/PKB inhibitor MK2206, 2 μM MEK1 inhibitor AZD6244 alone or in combination with 100 nM TZB or 6L1G (*P value versus MK2206 or as drawn by brackets in **h**, two-sided, unpaired Welch's *t*-test; Data in **i** are mean±s.e.m., *P value versus *ctrl* or as drawn in **i**, two-sided, unpaired Welch's *t*-test). Similar results were obtained with the AKT inhibitor GDC-0068.

Since ErbB2 remained continuously expressed in a panel of breast cancer cell lines treated with either agent (Fig. 1a–c; Supplementary Fig. 1A,B), and no substantial increase in internalization nor degradation of ErbB2 was observed (Supplementary Fig. 1C–F), we inspected the phosphostatus of ErbB2 as well as of ErbB3. Treatment with biparatopic DARPins reduced p-ErbB2 levels, as indicated both by a decline of total p-Tyr content in immunoprecipitated ErbB2 (Fig. 1a) and a progressive and uniform reduction of ErbB2 Tyr phosphorylation at specific tyrosine phosphorylation sites in the C-terminal tail of ErbB2 (Y1139, Y1169, Y1221/1222 and Y1248) and kinase domain (Y877) (Fig. 1b,c; Supplementary Fig. 1A,B) of ErbB2. By contrast, and in agreement with previous findings^3^,^10^, the treatment with trastuzumab did not affect total p-ErbB2 level (Fig. 1a) nor the individual p-Tyr sites (Fig. 1b,c; Supplementary Fig. 1A,B). Consequently, the recruitment of p-Tyr-binding adaptor proteins such as Grb2, propagating the cell survival and proliferation signals was impaired by 6L1G but not by the rather ineffective trastuzumab.

We found ErbB3 expression elevated under all treatment condition (Fig. 1a,b,d), probably as a result of transcriptional upregulation of ErbB3 by FoxO3a (*cf.* Discussion section). Nevertheless, p-ErbB3 levels declined instantly in response to ErbB2 blockade by both agents (Fig. 1d; Supplementary Fig. 1A,B), and in most ErbB2-depended cancer cells there was a permanent reduction of p-ErbB3. A slow re-phosphorylation of ErbB3 occurred solely in trastuzumab-treated SKBR3 cells (Fig. 1d), possibly due to autocrine ligand activation as reported earlier^10^. In conclusion, the dephosphorylation of ErbB3 resulting from ErbB2 blockade correlated with proliferation arrest, as seen for cytostatic compounds such as trastuzumab, whereas the induction of cell death also required a complete inhibition of ErbB2, as achieved by biparatopic DARPins (see below).

### Sustained inhibition of AKT is prerequisite for apoptosis

As shown in Fig. 1e, p-AKT level decreased rapidly upon treatment with 6L1G and trastuzumab, correlating with the fast dephosphorylation of ErbB3. Nevertheless, 6L1G caused more potent and, importantly, a sustained dephosphorylation of AKT. In contrast, trastuzumab induced a significant but transient AKT dephosphorylation, lasting up to 6–12 h in both BT474 and SKBR3 cells, and AKT became progressively re-phosphorylated thereafter. This p-AKT rebound occurred without re-phosphorylation of ErbB3 in most cell lines (Fig. 1b), hinting to an additional upstream stimulus feeding into the AKT pathway.

Since ErbB2 and EGFR are known to signal to the ERK pathway, we carried out time course analyses of p-ERK in response to receptor blockade. As shown in Fig. 1f, initiation of elevated p-ERK levels—notably in the absence of any elevated p-ErbB2—was detected 15 min after addition of trastuzumab or 6L1G. The underlying mechanism of this transient pulse is unclear, but it may be attributable to the suspended feedback regulation of RAF/MEK1/ERK leading to ERK stimulation, particularly in a low PTPase background^34^. Importantly, after 6–12 h of trastuzumab treatment, ERK phosphorylation returned to steady-state level in both BT474 and SKBR3 cells, while 6L1G further reduced the p-ERK level below the basal steady-state level, thus following the net decline of p-ErbB2.

To assess the relative contributions of the AKT and ERK pathways as safeguards against apoptosis upon ErbB2 blockade, we used selective kinase inhibitors as depicted in Fig. 1g. Note that AKT inhibition by MK2206, but not the MEK1/p-ERK inhibition by AZD6244, induced upregulation of ErbB3, again most likely by releasing the AKT-FoxO3a-ErbB3 negative feedback. As shown in Fig. 1h,i and Supplementary Fig. 1G, MK2206 exerted a cytostatic activity on BT474 cells, inducing G1 arrest without signs of apoptosis. Hence, the inhibition of AKT alone is not sufficient to elicit apoptosis in this model. On the other hand, inhibition of MEK1 by AZD6244 entirely failed to inhibit cell growth, but it enhanced significantly the anti-proliferative activity of MK2206. Nevertheless, even the combination of the two inhibitors could not trigger a full apoptotic response. However, a potent apoptotic response could be induced by combining MK2206 with trastuzumab, whereupon the p-ERK level remained normal (Fig. 1g,i; Supplementary Fig. 1G). By contrast, the combination with AZD6244 did not enhance the activity of trastuzumab, probably due to the inability to prevent the p-AKT rebound. Thus, the ERK pathway exerts merely a modulatory function and other survival pathways must also operate under the control of ErbB2/3 oncogenic unit. In contrast, the sustained inhibition of AKT seems to be the prerequisite for eliciting apoptosis in ErbB2-overexpressing tumours and signifies the importance of preventing the p-AKT rebound for the potency of anti-ErbB2/ErbB3-targeting agents.

### Intermolecular trapping of ErbB2 elicits cytotoxic response

**Figure 2:**
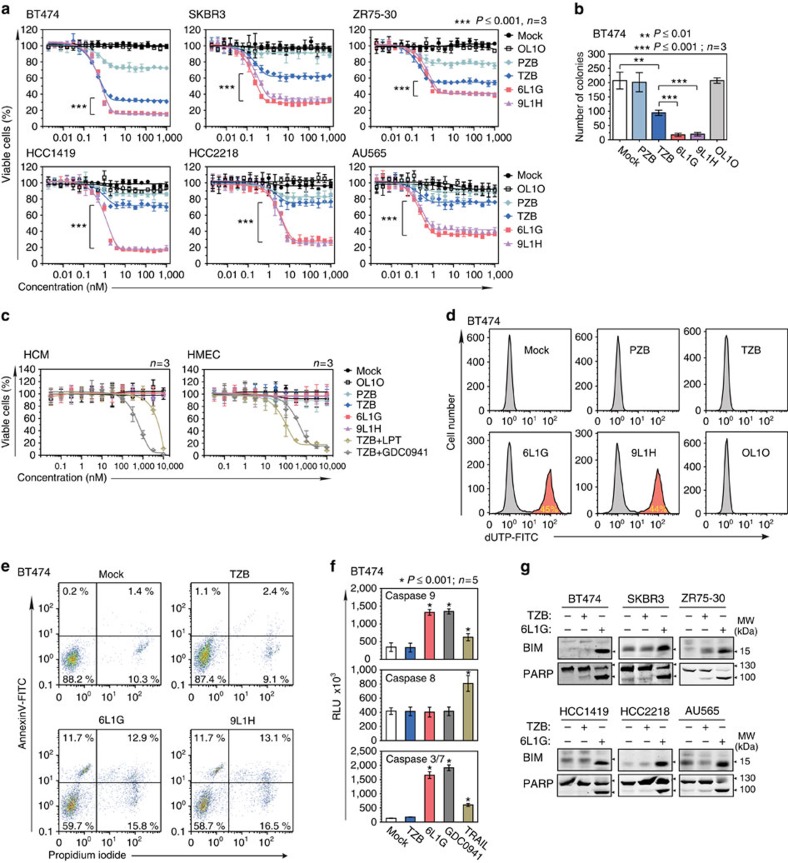
Induction of apoptosis upon trapping of ErbB2 by biparatopic agents. (**a**) Inhibition of proliferation of ErbB2-overexpressing breast cancer cell lines (XTT assay). Cells were treated for 96 h with 6L1G or 9L1H, non-binding DARPin control (OL1O), trastuzumab (TZB), pertuzumab (PZB) or PBS alone (mock); (*P value plateau 6L1G versus plateau TZB, two-sided, unpaired Student's *t*-test). (**b**) Colony growth of BT474 cells, treated for 96 h with 100 nM of indicated agents. The capacity of single cells to initiate colony outgrowth was determined by a clonogenic assay after 5-week re-culturing (two-sided, unpaired Student's *t*-test). (**c**) Proliferation of human cardiomyocytes (HCM) or embryonic human cardiomyocytes (HMEC) after 96 h of treatment (XTT assay). In addition to the same samples as applied in **a**, the cells were also treated with 100 nM TZB combined with lapatinib (LPT) and GDC-0941 at the concentrations indicated. (**d**) Induction of apoptosis as determined by TUNEL assay in histogram representation (apoptotic cells in red). Cells were treated with 100 nM of indicated agents for 72 h and analysed by FACS (Supplementary Fig. 2K). (**e**) Annexin V/propidium iodide (PI) assay of BT474 cells after 24 h treatment with 100 nM of indicated agents. Early apoptotic cells were monitored by Annexin V-FITC detection (upper left and right quadrants). Staining for membrane permeability (PI at 1 μg ml^−1^) indicates the late stages of cell death (upper and lower right quadrants). (**f**) Caspase activity in cell extracts from BT474 cells treated with 100 nM 6L1G or TZB for 72 h or 1 μM GDC-0941 or 10 ng ml^−1^ TRAIL for 12 h, determined by assays using specific chemiluminescent substrates for Caspase-3/7, 8 and 9 (*P value versus TZB, two-sided, unpaired Welch's *t*-test). (**g**) Analysis of apoptotic markers PARP cleavage (p89) and BIM expression after 72 h treatment with 100 nM of indicated agent.

To assess the cytotoxic activity of biparatopic DARPins *in vitro*, we treated a panel of ErbB2-overexpressing breast cancer cell lines (BT474, HCC1419, HCC2218, SKBR3, AU565 and ZR75-30) with wild-type PI3K activity and a range of EGFR, ErbB3 and PTEN expression levels (Fig. 2a). All cancer cell lines showed, without exception, the highest sensitivity to treatment with 6L1G and 9L1H, reflecting their cytotoxic activity. In contrast, application of trastuzumab resulted in an intermediate and highly variable cytostatic response, and pertuzumab only had minute effects on the growth of these refractory, ligand-independent ErbB2-overexpressing cell lines. The qualitative differences between biparatopic DARPins and trastuzumab in the induction of cell death, as determined by clonogenic assays (Fig. 2b), demonstrates the irreversible cancer cell damage inflicted solely by 6L1G or 9L1H.

The cytotoxic activity of the biparatopic DARPins was strictly dependent on a bivalent binding mode of both binding units simultaneously to two ErbB2 receptors, as deduced from the cell surface binding assays (Supplementary Fig. 2A,B), the dependency on orientation (Supplementary Fig. 2C,D) and the linker length (Supplementary Fig. 2E), indicating also the importance of the DARPin-induced change in the geometry of ErbB2_ECD. The anti-proliferative activity of all agents was dependent solely on ErbB2 binding, as shown by complete quenching of activity by addition of soluble ErbB2_ECD as competitor (Supplementary Fig. 2F). Importantly, the combination of either antibody with a biparatopic DARPin led to a reduction of the DARPins' cytotoxic activity (Supplementary Fig. 2G), indicating the importance of the DARPin-induced ErbB2 geometry for receptor inhibition.

Since the cytotoxic effects of the biparatopic agents are by design confined to tumour cells addicted to overexpression of ErbB2, non-tumour tissues with low ErbB2 expression should stay unaffected. We therefore tested the toxicity of DARPins on fetal human cardiac myocytes and normal human mammary epithelial cells. As shown in Fig. 2c, none of the DARPins impaired growth of these primary cells with low ErbB2 receptor levels, nor did the antibodies pertuzumab or trastuzumab. However, if trastuzumab was applied together with lapatinib, an EGFR/ErbB2 kinase inhibitor^5^ or with a PI3K inhibitor (GDC-0941), the combination treatment resulted in a substantial growth arrest or even an extensive cell death, depending on cell type. Hence, the small-molecule inhibitors appear to not only amplify the activity of low-potency antibodies on ErbB2-overexpressing tumours, but also exert toxicity on cells from tissues with low ErbB2 expression levels, leading inevitably to adverse side effects.

### Induction of G1 arrest and apoptosis upon ErbB2 blockade

The blockade of ErbB2 by 6L1G, 9L1H and trastuzumab, but not by pertuzumab, provokes cell cycle arrest in G1 phase in most ErbB2-overexpressing cell lines (Supplementary Fig. 2H). Examination of the master G1/S-phase transition regulators p27^KIP1^ and cyclin D1 revealed that their expression levels were influenced by both trastuzumab and 6L1G (Supplementary Fig. 1A), consistent with the recorded cell cycle profiles.

Importantly, the treatment with 6L1G and 9L1H, but none of the antibodies, yielded an additional peak with sub-G1 DNA content (Supplementary Fig. 2H,I), a typical hallmark of apoptosis. The quantification of cell populations with fragmented DNA in terminal deoxynucleotidyl transferase dUTP nick end labeling (TUNEL) assays revealed a marked accumulation of apoptotic cells after application of 6L1G and 9L1H, but not mAb treatments (Fig. 2d; Supplementary Fig. 2K). Consistent with these findings, Annexin V/propidium iodide staining demonstrated profound phosphatidylserine flipping proportional to the cytotoxic activity of the interrogated constructs (Fig. 2e). This early event in the execution of apoptosis has been also observed by time-lapse microscopy in BT474 cells treated with 6L1G, but not with trastuzumab (Supplementary Movies 1 and 2). Next, caspase activation was monitored, distinguishing between intrinsic and extrinsic induction of apoptotic pathways by inspecting initiator caspases 9 or 8, respectively. As shown in Fig. 2f, 6L1G or GDC-0941 (pictilisib), an inhibitor of PI3K, strongly induced caspase-9, while tumour necrosis factor-related apoptosis-inducing ligand (TRAIL) expectedly induced caspase-8, and the effector caspases-3/7 became activated by all apoptotic stimuli. We thus conclude that the cytotoxic activity of biparatopic DARPins, despite being *nota bene* extracellular ligands, is mainly attributable to activation of the intrinsic apoptotic pathway, and it occurs specifically in ErbB2-addicted tumour cells, as also suggested by the increased expression of the BH3-only pro-apoptotic Bcl-2-like protein 11 (BIM), a master regulator of apoptosis elicited by inhibition of EGFR family receptors^35^ (Fig. 2g).

### Efficient ErbB2 blockade induces apoptosis *in vivo*

**Figure 3:**
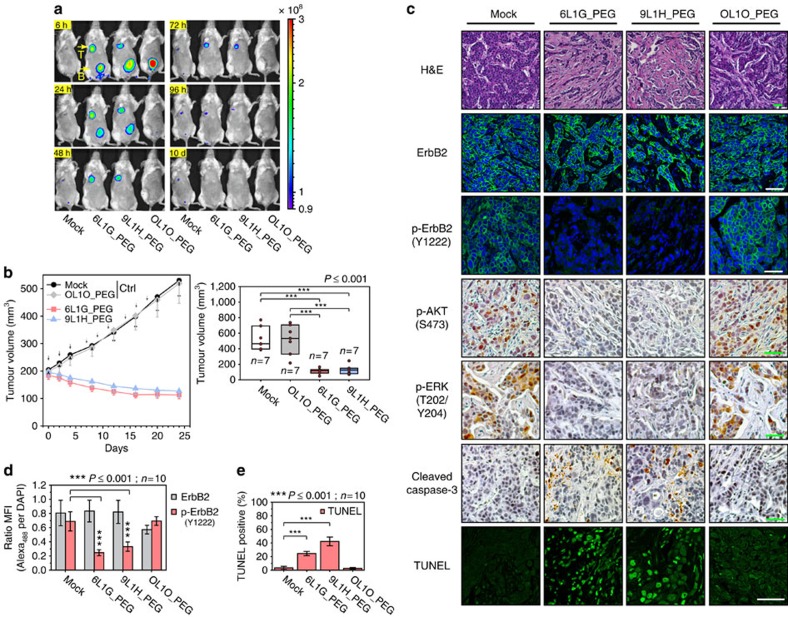
Sustained inhibition of ErbB2 signalling triggers apoptosis *in vivo.* (**a**) Biodistribution analysis in BT474-xenografted mice, injected with Alexa_680_-conjugated and PEGylated DARPins or PBS (i.v.; 1 mg kg^−1^). Mice were monitored on an IVIS imaging system for 10 days. Letters indicate tumour site (T) and bladder (B). (**b**) Tumour growth inhibition by PEGylated DARPins in SCID beige mice with pre-established BT474 xenografts. Left, treatment was started when tumours reached 200 mm^3^. Agents were injected three times per week (i.v.; 20 mg kg^−1^) as indicated by arrows. All error bars are s.e.m. Right, tumour size distribution of individual treatment groups after 24 d, for later time points see Supplementary Fig. 3C (two-sided, unpaired Students *t*-test). (**c**) *In situ* biomarker staining in treated BT474 xenografts. PFA-fixed, paraffin-embedded tumours were analysed by detecting the indicated cellular targets. Scale bars, 50 μm. (**d**) Per cent ErbB2 and p-ErbB2 (Y1222) staining intensity normalized to DAPI signal from ten tumour sections per treatment. ErbB2, p-ErbB2 and DAPI staining intensities were determined with ImageJ software (two-sided, unpaired Welch's *t*-test). (**e**) Per cent TUNEL-positive cells from ten tumour sections per treatment. BT474 tumours were stained with TUNEL and counterstained with DAPI, the total number of single cells was identified by DAPI using Cell Profiler software, and associated TUNEL signals were quantified and assigned above a threshold (70 units) as TUNEL-positive (two-sided, unpaired Welch's *t*-test).

The serum half-life of biparatopic DARPins was extended by N-terminal PEGylation^36^,^37^, which was shown to not affect their maximal *in vitro* activity (Supplementary Fig. 3A). To distinguish the DARPin-mediated anti-tumour activity from complement-dependent cytolysis or antibody-dependent cellular cytotoxicity responses, we have chosen the SCID beige mouse strain with a broad immunodeficiency profile and omitted fusions to carriers such as immunoglobulin Fc domains despite potentially better pharmacokinetics. The *in vivo* anti-tumour activity was analysed on BT474 xenografts that were inoculated orthotopically in the mammary fat pad of the mice. As shown in Fig. 3a, the targeted PEGylated constructs became efficiently enriched at the tumour site. Notably, both biparatopic DARPins caused a significant tumour regression (Fig. 3b), while the non-targeted PEGylated bivalent DARPin (vehicle control) and the phosphate-buffered saline (PBS) control did not show significant effects. Even though complete tumour remission could not be achieved and post-treatment tumour regrowth has occurred in most animals, attributable to insufficient half-life extension by PEGylation, the anti-tumour activity of biparatopic DARPins *in vivo* was evident (Supplementary Fig. 3B,C).

Hematoxylin and eosin staining of sections from the regressing DARPin-treated tumours revealed large areas of tumour destruction replaced by fibrous myxoid stroma (Fig. 3c). As shown in Fig. 3c,d, IHC analysis of the tumours revealed no significant difference in the average ErbB2 expression level per cell among treatments, while ErbB2 phosphorylation was found significantly reduced after administration of active DARPins. In line with this observation, the examination of downstream protein kinases confirmed diminished p-AKT and p-ERK staining in the successfully treated tumours (Fig. 3c). Tumour growth regression correlated with the ability to induce apoptosis *in vivo*, as manifested by significantly stronger TUNEL staining (Fig. 3c,e), and enrichment of active caspase-3 in the treated tumours. These data confirm that DARPins retained the ability to induce apoptosis *in vivo*, and thus, the achieved tumoricidal activity was attributable to the same mode of action as in the tissue culture tumour models. Moreover, measurements of the downstream kinases indicate that the DARPin-induced apoptosis likely employs the same mechanism as observed *in vitro* (see above).

### Efficient ErbB2 blockade induces apoptosis in 3D tumour models

**Figure 4:**
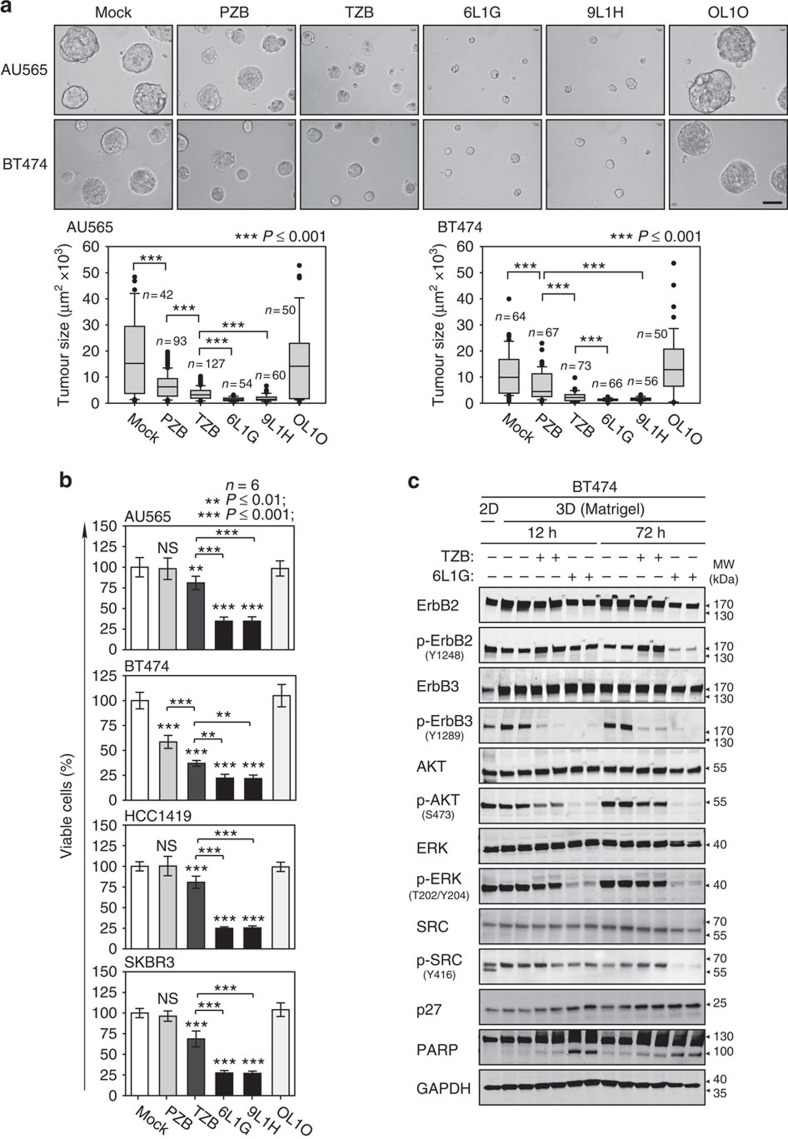
Treatment efficacy in 3D tumour spheroid model. (**a**) 3D spheroid cultures of cancer cells grown on Matrigel. Top, micrographs showing AU565 and BT474 spheroids after 12 days with indicated treatment, for abbreviations see Fig. 2a. Bottom, size distribution of treated spheroids. Ten micrographs were analysed with Image J software (Mann–Whitney test). Scale bar, 50 μm. (**b**) Proliferation of treated 3D cultures of AU565, BT474, SKBR3 and HCC1419 in Matrigel were analysed by a modified XTT assay (*P value versus mock or as drawn, two-sided, unpaired Welch's *t*-test). (**c**) AKT re-phosphorylation in 3D spheroids. BT474 cultures on Matrigel were treated by TZB or 6L1G and whole-cell extracts were analysed by immunoblots.

Maintaining the breast cancer cell lines in three-dimensional (3D) cell culture alters proliferation and morphology characteristics and may affect sensitivity to ErbB2 blockade. In 3D cultures of BT474 and AU565 cells on a laminin-rich reconstituted basement membrane (Matrigel), the tumour cells become organized into acini-like solid spheroid structures resembling a ductal glandular like architecture^38^. As shown in Fig. 4a, both DARPins and, to a lesser extent, trastuzumab abrogated the 3D growth of BT474 and AU565 spheroids. The proliferation was determined also by 3D XTT assays (Fig. 4b), and we found that 6L1G showed consistently stronger effects than trastuzumab in all tested ErbB2-overexpressing cell lines in 3D cultures. We further investigated whether the signalling effects of ErbB2 blockade observed in 2D culture were equivalent also in a 3D matrix. As shown in Fig. 4c, p-ErbB3 dephosphorylation was observed after trastuzumab and 6L1G treatment, as was the subsequent dephosphorylation of downstream p-AKT. Importantly, the p-AKT rebound was observed 72 h after trastuzumab treatment, similar to the 2D analysis, in the absence of p-ErbB3 upregulation. In contrast, 6L1G markedly blocked p-ErbB2 and p-AKT rebound after 72 h. Likewise, marked induction of apoptosis after 6L1G treatment or the trastuzumab/MK2206 combination (but not trastuzumab alone) was observed under the 3D cell culture conditions.

### Inhibition of ErbB2–ErbB3 heterodimerization

**Figure 5:**
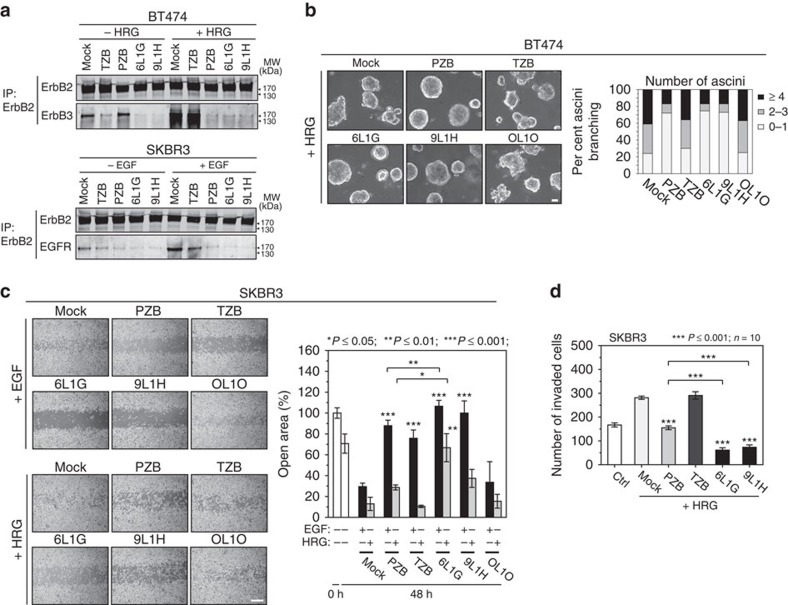
Pan-ErbB2 inhibition abolishes heterodimerization of ErbB2 irrespective of the presence of ligand. (**a**,**b**) Inhibition of ligand-dependent and ligand-independent ErbB2 heterodimers. After preincubation with 100 nM of the anti-ErbB2 agents, ErbB2–ErbB3 dimerization was triggered with 1 nM heregulin-β1 (HRG) in BT474 cells (top) and ErbB2–EGFR dimerization with 4 nM epidermal growth factor (EGF) in SKBR3 cells (bottom). Receptors were crosslinked with reducible crosslinker DTSSP. (**a**) ErbB2 was immunoprecipitated by anti-ErbB2-C-terminal antibody, and the co-immunoprecipitated receptor was determined by immunoblot analysis. (**b**) Micrographs show HRG-induced morphogenesis (acini branching) after 12 days of co-treatment (top). Spheroid branching was quantified by three times scoring of 100 acini classified into three populations (bottom), depending on the number of buds per acinus. Scale bar, 50 μm. (**c**) Effect of the ErbB2-targeting agents on cell migration. SKBR3 cells were seeded to confluence, and scratch wounds of 500 μm were made in the monolayers. Cells were treated with the indicated agents (100 nM) and co-stimulated with 4 nM EGF or HRG. Left, representative micrographs were taken after 48 h. Right, the cell migration was expressed as per cent open area relative to the area at time point 0 h (*P value versus respective mock (EGF/HRG) or as drawn, two-sided, unpaired Welch's *t*-test). Scale bar, 500 μm. (**d**) Effect of the ErbB2-targeting agents on cell invasion induced by HRG. SKBR3 cells pre-seeded on Matrigel in a Boyden-type invasion chamber were treated with the indicated agents (100 nM) and co-stimulated for 72 h with 1 nM HRG. Data are mean±s.e.m. (*P value versus mock (+HRG) or as drawn, Mann–Whitney test).

The trapping of ErbB2 by biparatopic agents was proposed to obstruct the canonical interactions with ErbB3 or EGFR^31^. We therefore tested this notion by inducing ErbB2–ErbB3 heterodimerization with heregulin-β1 (HRG) in pretreated BT474 cells and performed receptor crosslinking on the cell surface with thiol-cleavable 3,3′-dithiobis[sulfosuccinimidylpropionate] (DTSSP). Co-immunoprecipitation revealed that DARPins interfered with the HRG-induced ErbB2–ErbB3 heterodimerization to a similar extent as pertuzumab (Fig. 5a). Moreover, the ligand-independent heterodimerization of ErbB2–ErbB3, occurring on and driving growth of ErbB2-overexpressing tumours^3^, was also abrogated by DARPins and by trastuzumab, thereby inducing dephosphorylation of ErbB3 by uncoupling it from the ErbB2-mediated transphosphorylation (Fig. 1a,b,d).

Analysis of ErbB2–EGFR heterodimerization was performed analogously with the corresponding ligand (EGF) on SKBR3 cells, which express higher levels of EGFR. As shown in Fig. 5a, 6L1G and 9L1H reduced likewise the ErbB2–EGFR heterodimerization irrespective of the presence of EGF. Altogether, while anti-ErbB2 mAbs bring about selective blockade of distinct ErbB2 heterodimer complexes, biparatopic DARPins achieve a pan-ErbB2 inhibition, disengaging the trapped receptors from all productive homo- and heterodimer interactions.

Next, we examined the effects of ErbB2-targeting agents on the morphogenesis in 3D culture, a process that is known to be under the control of ErbB2-containing heterodimers. As shown in Fig. 5b, BT474 spheroids underwent morphological changes upon addition of HRG, manifested by pronounced budding/branching and invasion of the surrounding matrix. Again, this phenotype was robustly inhibited by 6L1G and 9L1H, as well as by pertuzumab, whereas trastuzumab had no effect.

We then investigated the effect of DARPins on cell motility in a wound-healing assay with SKBR3 cells, high in both EGFR and ErbB3. As shown in Fig. 5c, both EGF and, in particular, HRG induced an extensive resealing of scratches in cell monolayers after a 2-day treatment. The EGF- or HRG-induced cell migration was reversed by 6L1G and 9L1H but only retarded by the mAbs, correlating with the specific ability of each agent to disrupt the ErbB2 heterodimers (Fig. 5a). Last, a cell invasion assay using SKBR3 cells stimulated with 1 nM HRG revealed that biparatopic agents reduced the number of invading cells more strongly than pertuzumab, which nonetheless neutralized the effect of HRG (Fig. 5d).

### Interplay between ErbB2 and ErbB3 in apoptosis induction

**Figure 6:**
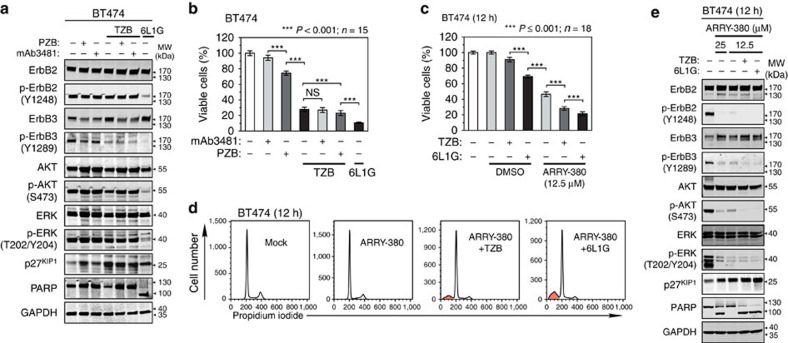
Blockade of ErbB3 receptor is not sufficient to induce apoptosis without inhibition of ErbB2. (**a**,**b**) ErbB2-overexpressing cells do not utilize ligand stimulation for reactivation of AKT after inhibition of ErbB3 phosphorylation. BT474 cells were treated for 72 h with 100 nM 6L1G, PZB and anti-ErbB3 antibody mAb3481, combined with TZB or alone. The status of ErbB2 and ErbB3 receptors and the downstream effectors was determined by immunoblot analysis (**a**), and effects of the treatments on proliferation were estimated by XTT assays (**b**) (Supplementary Fig. 4A,B) (two-sided, unpaired Welch's *t*-test). (**c**–**e**) Combination of ErbB2 and ErbB3 blockade is required to prevent p-AKT rebound and induce cell death. Inhibition of XTT cell proliferation (**c**), cell cycle (**d**) and receptor signalling (**e**) after 12 h of treatment with the ErbB2-selective kinase inhibitor ARRY-380 alone or in combination with 100 nM TZB or 6L1G (two-sided, unpaired Student's *t*-test).

In a simplified view, the proliferation of ErbB2-overexpressing tumour cells is driven predominantly by both ligand-independent homodimerization of ErbB2 (ref. 4) and heterodimerization with unliganded ErbB3 (ref. 3), whilst an autocrine ligand stimulation^10^ has been proposed to confer resistance against ErbB2 blockade via formation of ligand-containing heterodimers. We used pertuzumab and the anti-ErbB3 antibody mAb3481, which abolishes HRG-induced growth (Supplementary Fig. 4A), in combination with trastuzumab to test whether increased ligand stimulation accounts for the reactivation of AKT after trastuzumab treatment. As shown in Fig. 6a,b, neither pertuzumab nor mAb3481 significantly blocked the p-AKT rebound or potentiated the trastuzumab activity to the level of 6L1G (Supplementary Fig. 4B), nor did they induce downregulation or dephosphorylation of ErbB2. We thus conclude that the capability to induce apoptosis in BT474 cells does not rely on blocking ligand-induced receptor heterodimerization. Instead, it is inherent in the obstruction of ErbB2 from participating in non-liganded homo- or heterodimers, which are promoted by ErbB2 overexpression.

To evaluate the relative contribution of ErbB2 kinase activity to AKT activation, we used the selective ErbB2 inhibitor ARRY-380. Since a complete ablation of ErbB2 function, for example, by gene knockdown, is known to be lethal in ErbB2-dependent tumours^8^, we deliberately used sublethal inhibitor concentrations to study its effects on downstream signalling (Fig. 6c–e). As shown in Fig. 6e, ARRY-380 diminished p-ErbB2 levels by >90%. Under these conditions, p-ErbB3 and p-AKT levels were markedly reduced, yet residual phosphorylation could be detected. Importantly, the addition of trastuzumab to pretreated cells induced a complete abolition of p-ErbB3 and p-AKT, accompanied by an extensive cell death (Fig. 6d), reminiscent of 6L1G treatment in the absence of ARRY-380. These findings further underline the importance of simultaneous inhibition of ErbB2 activity and the obstruction of ErbB2–ErbB3 heterodimerization, which are apparently two different factors that are both required for achieving a full apoptotic response.

**Figure 7:**
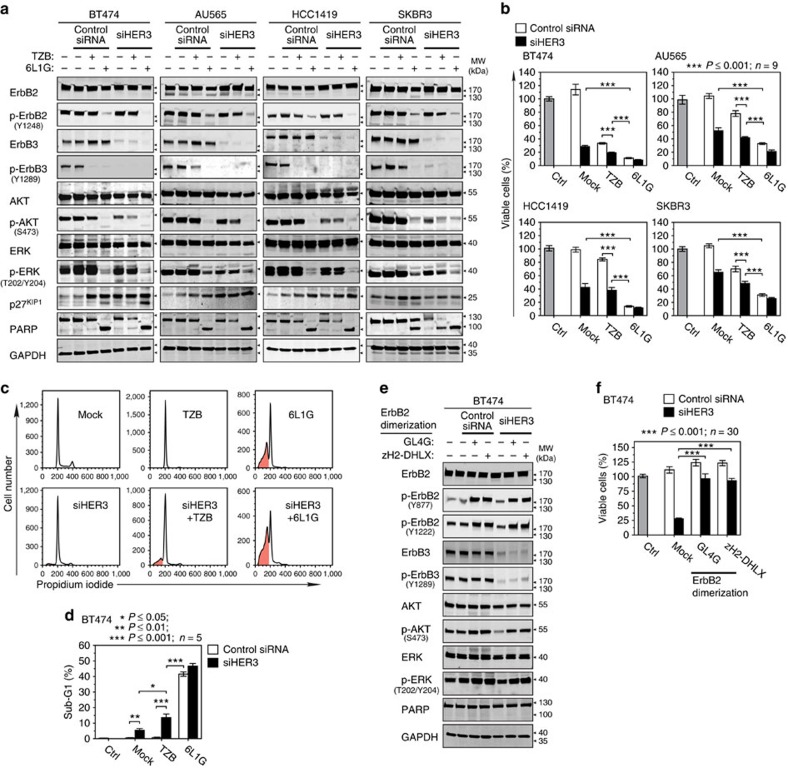
ErbB3 knockdown is not sufficient to induce apoptosis without inhibition of ErbB2. (**a**–**d**) Phosphorylated ErbB2 in the absence of ErbB3 receptor expression correlates with AKT reactivation and is sufficient to suppress apoptosis in ErbB2-dependent cancer cells. Inhibition of receptor signalling (**a**), proliferation (**b**), cell cycle (**c**) and apoptosis (**d**) 72 h after ErbB3 knockdown by siHER3 alone or in combination with 100 nM TZB or 6L1G (Supplementary Fig. 4C–F) (*P value in **b** Mann–Whitney test; data in **d** are mean±s.e.m.,*P value in **d** two-sided, unpaired Student's *t*-test). (**e**,**f**) Stimulation of p-AKT (**e**) and restoration of cell proliferation (**f**) in BT474 cells with simultaneous ErbB3 knock down and hyperactivation of ErbB2 homodimers with 50 nM of GL4G (G3-L4-G3) or zH2-DHLX (zHer2-L2-DHLX). Homo-bivalent GL4G or zH2-DHLX stimulate ErbB2 dimerization and consequently ErbB2 phosphorylation and downstream signalling to p-AKT (**e**) and finally rescue the growth inhibitory effect of siHER3 in XTT assays (Mann–Whitney test).

We next carried out a knockdown of the ErbB3 receptor by RNA interference. As shown in Fig. 7a, siHER3 substantially attenuated ErbB3 expression and, consequently, p-ErbB3 levels. Notably, AKT still remained partially phosphorylated in siHER3-treated cells, reminiscent of the p-AKT rebound in the presence of de-phosphorylated ErbB3 upon trastuzumab treatment (Figs 1b and 4c). Consequently, ErbB3 knockdown does not induce apoptosis efficiently in these cell lines (Fig. 7b–d; Supplementary Fig. 4C–E) and, despite strongly impeding cell proliferation, it did not achieve the full cytotoxic activity of 6L1G. Nevertheless, it showed a synergistic effect when combined with the concomitant inhibition of ErbB2 (Supplementary Fig. 4F).

These findings prompted us to analyse whether there is an ErbB3-independent activation of the AKT pathway downstream of ErbB2, even though ErbB2 itself does not possess p85 PI3K docking sites. Therefore, we constructed homo-bivalent binding agents GL4G (G3-L4-G3) or zH2-DHLX (zHer2-L2-DHLX), directed either against ErbB2 ECD_4 or ECD_3, which potently hyperactivated ErbB2 by promoting its homodimerization (Fig. 7e). We found that both agents stimulate formation of p-ErbB2 (Y877 and Y1222), p-AKT and p-ERK. Intriguingly, in combination with knockdown of ErbB3, both GL4G and zH2-DHLX rescued the p-AKT decrease, indicating the existence of an alternative (ErbB3-independent) route linking ErbB2 receptor to p-AKT activation. Consistently, growth inhibition by siHER3 was rescued almost completely by both ErbB2 homodimerization agents (Fig. 7f). Thus, both p-ErbB2 and p-ErbB3 are—despite their mutual interdependence—individually contributing to the survival of treated cancer cells by causing reactivation of AKT.

### Small-GTPase RAS links ErbB2 with PI3K/AKT pathway

**Figure 8:**
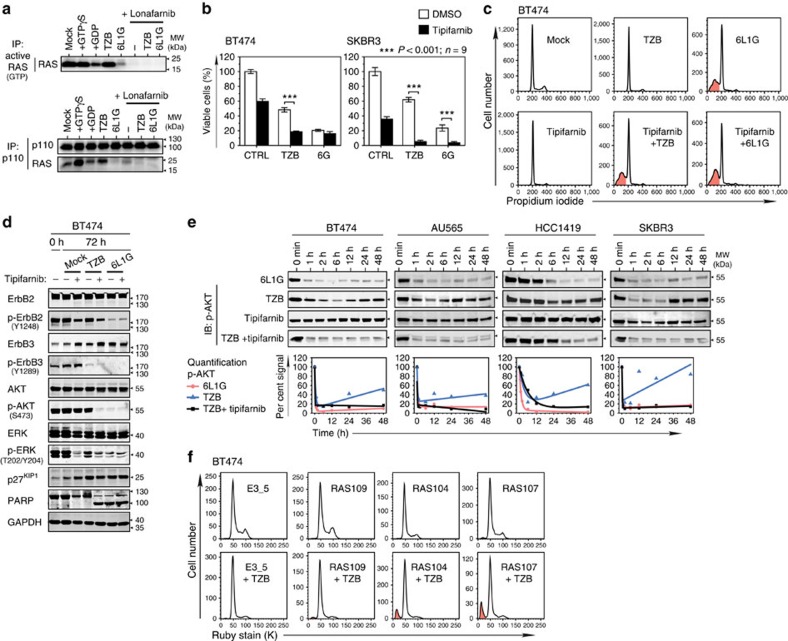
RAS-PI3K crosstalk mediates AKT reactivation in the absence of p-ErbB3. (**a**) 6L1G but not TZB treatment blocks both RAS-GTP formation and RAS–p110α interaction. RAS-GTP pull-down assay using GST-RBD beads (top) and co-immunoprecipitation of p110α and RAS with pan-RAS immunoblot detection (bottom) after 72 h of treatment of BT474 cells with 5 μM of RAS farnesyltransferase inhibitor lonafarnib alone or in combinations with 100 nM TZB and 6L1G. (**b**–**e**) Combination treatment of TZB with RAS farnesyltransferase inhibitor blocks p-AKT rebound and triggered apoptosis. Inhibition of XTT cell proliferation (**b**), cell cycle (**c**), and downstream signalling (**d**) after 72 h of treatment with 5 μM tipifarnib in combination with 100 nM TZB or 6L1G (Supplementary Fig. 5G). The p-AKT rebound was quantified over 48 h in **e**. In **b**–**e**, tipifarnib was used at 5 μM concentration in BT474, AU565, HCC1419 and at 1 μM in SKBR3 cells. Note that ERK phosphorylation was inhibited by both 5 μM tipifarnib and lonafarnib (two-sided, unpaired Welch's *t*-test). (**f**) Selective RAS inhibitors RAS104 and RAS107, blocking the RAS-RBD binding interface, induce apoptosis in combination with TZB after 3 days treatment in BT474 cells. The non-interfering RAS binder RAS109 or the non-targeted DARPin E3_5 were used as controls with minute or no pro-apoptotic activity, respectively. BT474 cells were transfected with plasmids expressing RAS104, RAS107, RAS109 or E3_5 GFP-fusions and treated with 100 nM TZB 24 h later (Supplementary Fig. 5B). The PFA-fixed cells were stained with Vybrant Ruby stain, and the transfected GFP-positive population was analysed by FACS for cell cycle distribution.

As discussed above, ErbB2 dephosphorylation and dissociation of Grb2 coincided with 6L1G-induced apoptosis (Fig. 1a). Since Grb2 promotes SOS-mediated GDP/GTP exchange, we estimated RAS activity by specifically detecting the GTP-bound form of RAS in a GST-RBD (RAS-binding domain) pull-down assay. As shown in Fig. 8a, 6L1G inhibited RAS in BT474 cells to a similar extent as the farnesyltransferase (FT) inhibitor lonafarnib, a potent blocker of RAS function. Trastuzumab did not suppress RAS-GTP, owing to the inefficient blockade of ErbB2. Active RAS is known to directly interact and promote PI3K p110α activity independently of p85 in some tumours^39^. 6L1G and lonafarnib effectively impaired the interaction of RAS with p110α, whereas RAS–p110α complex formation was even stimulated by trastuzumab and correlated with the relative increment of p-AKT (Fig. 8a). Thus, the ErbB2/Grb2/SOS/RAS pathway may contribute to the activation of PI3K/AKT in the absence of ErbB3 and, consequently, to the rebound of AKT activity in the presence of phosphorylated ErbB2, as it occurs under the conditions of incomplete ErbB2 blockade by trastuzumab. Furthermore, since PI3K integrates both the ErbB2/Grb2/SOS/RAS and ErbB3/p85 pathway, the ErbB2-overexpressing cancer cells become addicted to the PI3K signalling hub, as manifested by increased susceptibility to PI3K inhibitors (Supplementary Fig. 5A), compared with ErbB3 inhibition (Fig. 7b).

Consistent with these findings, lonafarnib as well as tipifarnib, another RAS FT inhibitor, potentiated the cytotoxic activity of trastuzumab (Fig. 8b), and the RAS inhibitor/trastuzumab combination became as efficient in inducing apoptosis as the biparatopic agents (Fig. 8c). Importantly, the gain of cytotoxic activity directly correlated with the inhibition of AKT re-phosphorylation (Fig. 8d,e). Since tipifarnib as single agent did not substantially influence p-AKT levels in ErbB2-overexpressing cells, we deduce that the synergistic inhibition of p-AKT by tipifarnib and trastuzumab was the consequence of concurrent decoupling of AKT from p-ErbB3/p85/p110 by trastuzumab together with RAS/p110 inhibition by tipifarnib.

**Figure 9:**
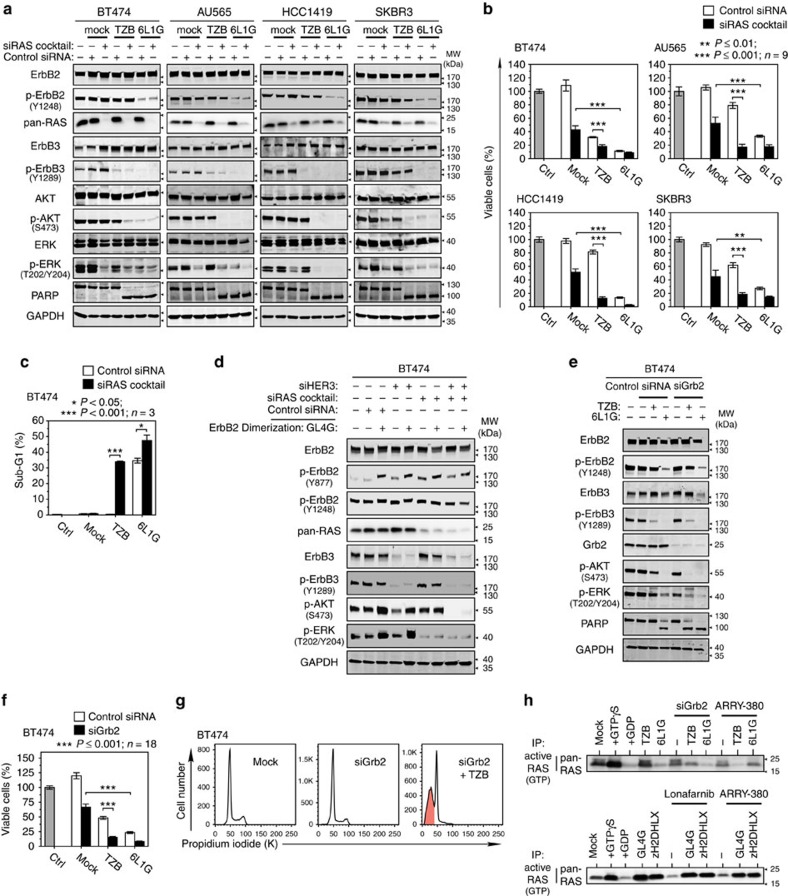
ErbB2 homodimers stimulate RAS-mediated AKT reactivation. (**a**–**c**) HRAS/KRAS knockdown in combination with TZB was sufficient to abolish the p-AKT rebound (**a**), to block proliferation (**b**) and to induce apoptosis (**c**) *cf*. (Supplementary Fig. 5E–H). Analysis was done 72 h after knockdown of HRAS/KRAS by siRNA cocktail in combination with 100 nM of TZB or 6L1G (**a**–**c**). Note that HRAS/KRAS knockdown robustly blocks p-ERK in the presence of an active ErbB3/PI3K/AKT pathway (*cf*. Supplementary Fig. 5G) (*P value in **b** Mann–Whitney test; data in **c** are mean±s.e.m.,*P value in **c** two-sided, unpaired Student's *t*-test). (**d**) ErbB2 homodimers depend on HRAS/KRAS expression to mediate the p-AKT rebound in the absence of ErbB3 expression. Double knockdown of ErbB3 and RAS results in effective p-AKT and p-ERK inhibition and blocks GL4G-induced p-AKT and p-ERK stimulation completely after 72 h of treatment. (**e**–**g**) Grb2 knockdown in combination with TZB-induced apoptosis by inhibition of the p-AKT rebound. BT474 cells were treated for 72 h with 100 nM TZB or 6L1G in combination with Grb2 knockdown by siRNA. Immunoblot detection (**e**), XTT cell proliferation assays (**f**) and cell cycle (**g**) (*P value in **e** Mann–Whitney test). (**h**) Detection of RAS-GTP by pull-down assay with GST-RBD. BT474 cells were treated for 72 h with 100 nM TZB or 6L1G in combination with siGrb2 knockdown or 12.5 μM ARRY-380 (top), or 50 nM GL4G or zH2-DHLX in combination with either 5 μM lonafarnib or 12.5 μM ARRY-380 (bottom).

To validate the significance of RAS—p110α interaction for the crosstalk between p-ErbB2 and PI3K, we made use of intracellular RAS-binding proteins (Supplementary Fig. 5B), either blocking the RAS-RBD binding interface (RAS104, RAS107) or not interfering with the interaction with p110 (RAS109). Accordingly, the cells carrying RAS104 and RAS107 showed significantly higher susceptibility to trastuzumab treatment than the cells with no RAS interference (Fig. 8f). Furthermore, we silenced RAS expression using siRNAs against the two prevailing RAS isoforms HRAS and KRAS^39^. As shown in Fig. 9a and Supplementary Fig. 5C, the siHRAS/siKRAS cocktail efficiently downregulated pan-RAS expression without substantial reduction of p-AKT (except for SKBR3 cells, probably due to their high EGFR). Note that HRAS/KRAS knockdown did not only block p-ERK, but it also induced p-ErbB3 upregulation, possibly by interfering with the AKT-FoxO3a-negative feedback. Importantly, trastuzumab induced, in the background of suppressed RAS expression, a sustained decrease of p-AKT, despite the incomplete blockade of ErbB2. Consequently, RAS knockdown substantially potentiated the anti-proliferative effect of trastuzumab-induced ErbB3 inhibition (Fig. 9b; Supplementary Fig. 5D) and elicited extensive cell death in all examined cell lines (Fig. 9c, Supplementary Fig. 5E–H).

In agreement with these observations, the combination of selective double knockdown of both ErbB3 and RAS by siRNA treatment was sufficient to block the p-AKT rebound occurring after single ErbB3 knockdown (Fig. 9d). Furthermore, hyperactivation of ErbB2 homodimers by GL4G, which is sufficient to reactive p-AKT in the absence of ErbB3, remained without effect upon knockdown of RAS (Fig. 9d), thereby placing RAS between ErbB2 and p-AKT.

Next we examined the direct linkage between ErbB2 and RAS by selectively downregulating the adaptor protein Grb2 (Fig. 9e–g). The combination of siGrb2 and trastuzumab treatment prevented p-AKT rebound and, consequently, blocked proliferation, induced cell cycle arrest and apoptosis. The RAS pull-down assay revealed that siGrb2 as well as ARRY-380 treatment, particularly in combination with trastuzumab or 6L1G, led to a decline of the active form of RAS (Fig. 9h). In contrast, RAS-GTP was induced upon homodimerization and hyperphosphorylation of ErbB2; both GL4G and zH2-DHLX potently stimulated active RAS, compensated the effect of lonafarnib and ARRY-380 on RAS inhibition and gave rise to an overall decrease of efficacy of RAS, ErbB2 and AKT inhibition.

In summary, we found that in ErbB2-overexpressing breast cancer cells the ErbB2/Grb2 interaction leads to the direct activation of RAS which, in turn, mediates via interaction with p110α the p-AKT rebound in response to an incomplete blockade of ErbB2. Thus, we conclude that both routes to PI3K activation, emanating from ErbB2/Grb2/RAS and ErbB3, must be interrupted to achieve full anti-tumour response.

Finally, we investigated whether the described principles underlying apoptosis induction after ErbB2/3 blockade shares a common signalling mechanism with anoikis, a specific type of cell death occurring during organogenesis of normal mammary epithelium. Anoikis is known to be reverted by pathological overexpression of ErbB2 (ref. 40) in conjunction with hyperactivation of protein kinase Src, which may directly interact with ErbB2 (ref. 41) and engage the Src/Erk/BIM survival pathway^42^. To examine a potential involvement of Src, we made use of selective Src inhibitors, siSRC or a dominant-negative form of Src in 2D and 3D cultures of BT474 cells (Supplementary Fig. 6A–D). Blockade of ErbB2/3 by the biparatopic DARPin consistently attenuated Src activity (Fig. 4c); conversely, the inhibition of Src alone or in combination with trastuzumab does not trigger apoptosis in the 3D tumour model and does not induce PARP cleavage. Accordingly, Src inhibition does not reduce p-AKT nor prevents p-AKT rebound upon trastuzumab treatment in the models investigated here.

In stark contrast, apoptosis also progressed in the presence of active Src under conditions of simultaneous RAS inhibition and an incomplete ErbB2 blockade (achieved by coincubation with tipifarnib and trastuzumab (Supplementary Fig. 6A)). In accordance, expression of constitutively active Src did not lead to elevated p-AKT levels after the inhibition of ErbB3 and RAS by the trastuzumab/tipifarnib combination or by 6L1G (Supplementary Fig. 6E–G). Moreover, the Co-IP analysis of ErbB2–Grb2 and ErbB2–Src complexes under several treatment conditions revealed that Src inhibition did not affect integrity of the ErbB2–Grb2 complexes and only had a small effect on the phosphorylation of ErbB2 (Supplementary Fig. 6H).

Altogether, this argues for the existence of two distinct apoptotic programmes that are executed (i) upon complete ErbB2 blockade in ErbB2-addicted tumours and (ii) during anoikis in the development of normal mammary epithelium without ErbB2 addiction. The latter employs indeed primarily the Src/Erk/BIM axis. By contrast, in ErbB2-addicted tumours induction of apoptosis occurs independently of Src status. In conclusion, as shown in this study, apoptosis necessitates complete and sustained blockade of the PI3K/AKT pathway for preventing p-AKT rebound, and thus requires an intervention at both the ErbB3/p85 and the ErbB2/RAS/p110 routes feeding into PI3K/AKT.

## Discussion

Although trastuzumab- and TKI-treatment provide clinical benefit with respect to improved progression-free survival, the persisting problem in therapy is that even patients who initially respond to these substances invariably develop resistance while progressing towards advanced disease^23^. Antagonistic monoclonal antibodies and small-molecule inhibitors of receptor tyrosine kinases or intracellular pro-survival pathways can lower the apoptotic threshold and thus increase the tumour-specific cytotoxicity of chemotherapeutics. Nevertheless, an effective treatment of ErbB2-overexpressing breast tumours necessitates strategies that not only rely on rapid induction of cell death, but also counter the tumour escape mechanisms leading to a long-term adaptation to the treatment. Therefore, such agents should ideally interfere with the multiple compensatory mechanisms that would otherwise minimize the drug potency by generating a new steady state of the oncogenic network^27^. By targeting two distinct epitopes of ErbB2 with appropriately designed biparatopic binding DARPins^31^ that completely disengage ErbB2 from its signalling network, we developed powerful agents with cytotoxic activity against ErbB2-addicted breast cancer cells and no measurable effects on normal cells with physiological ErbB2 levels. The cytotoxic activity was overall retained in cells with an additional p53 deficiency or low PTEN expression and even in cells bearing *PIK3CA* activating mutations, these biparatopic anti-ErbB2 agents still displayed significant anti-proliferative activity, although the pro-apoptotic activity has been significantly reduced, indicating the central role of PI3K/AKT pathway in mediating resistance to ErbB2 blockade.

**Figure 10:**
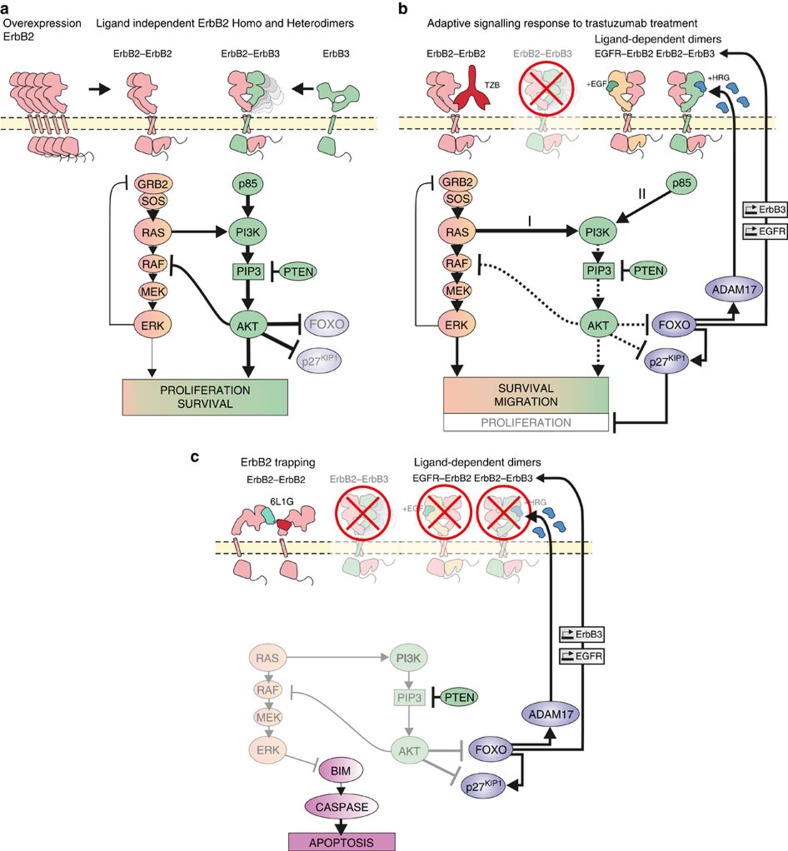
Model of induction of apoptosis and adaptive resistance in response to ErbB2 blockade. (**a**) Overexpression of ErbB2 is sufficient to activate ErbB2 and ErbB3 in the absence of ligands and drives tumour growth predominantly via the ErbB2/ErbB3/PI3K/AKT signalling axis. This signalling route constitutively suppresses negative feedback regulation between AKT and ErbB3, which is however relieved upon blockade of ErbB2/3 receptors or the downstream signalling pathways. (**b**) Trastuzumab treatment induces partial ErbB2 blockade by selectively interfering with the ligand-independent ErbB2/ErbB3 heterodimers, thereby uncoupling ErbB3 from PI3K/AKT reactivation. Here we identified a novel adaptation response that emanates from ErbB2/RAS and bypasses ErbB3 for the activation of PI3K/AKT signalling. (**c**) Biparatopic DARPins obstruct all ligand-dependent and ligand-independent complexes of ErbB2. Such a pan-ErbB2 inhibition blocks PI3K/AKT signalling cascade and consecutive adaptive responses, leading to a stable OFF state of the ErbB oncogenic network. Consequently, intrinsic apoptosis is induced which prevents emergence of adaptive resistance.

For optimal antitumor effects of therapeutic agents acting on overexpressed ErbB2, a sustained and complete inhibition of ErbB3 phosphorylation and the downstream signalling to PI3K/AKT is required. As a consequence of the relieved negative feedback loop operating in tumours with elevated PI3K/AKT signalling, however, there is typically upregulation and marked increase of ErbB3 in the plasma membrane, accompanied by re-phosphorylation of ErbB3 due to residual ErbB2 activity^22^,^43^. Thus, ErbB3 signalling is ‘buffered' as long as inhibition of the ErbB2 kinase is incomplete, and this applies particularly to the small-molecule kinase inhibitors. Such adaptive capabilities of the ErbB oncogenic signalling network reveal fundamental limitations of monotherapy for inhibiting feedback-regulated pathways (Fig. 10a–c). Therefore, alternative strategies are required to silence oncogenic ErbB2/3 signalling effectively.

In the present study, we describe a new adaptive response leading to reactivation of PI3K/AKT signalling during trastuzumab treatment, which occurs even under circumstances when ErbB3 remains de-phosphorylated as a consequence of ErbB2 blockade (Fig. 10b). This signalling pathway, bypassing ErbB3, operates in the majority of examined ErbB2-overexpressing breast cancer cell lines, and it is clearly distinct from other mechanisms of adaptive resistance to trastuzumab such as upregulation of ErbB family receptors and cognate RTKs, or increased expression of the respective ligands^27^,^44^. We showed that PI3K/AKT evades sustained inhibition, because trastuzumab fails to induce dephosphorylation of ErbB2 itself. Notably, the lack of ErbB2 dephosphorylation had been previously postulated to be one of the key reasons why a longstanding trastuzumab treatment leads to development of acquired resistance in ErbB2-addicted cells^10^. Yet the precise compensatory mechanisms leading to reactivation of PI3K/PKB in the absence of functional ErbB2–ErbB3 complexes had not been understood.

In fact, we have now identified the ErbB2–RAS interaction as a crucial regulatory switch determining whether the AKT pathway becomes inhibited persistently upon ErbB2/3 targeting or only transiently with a subsequent reactivation. The activation of the PI3K pathway via RAS is well characterized; Grb2, an adaptor protein binding the phosphorylated tail of ErbB2, associates with SOS, which then activates RAS that, in turn, triggers allosteric activation of p110α independently of p85 (ref. 45). Indeed, p110α is a critical effector for RAS-driven tumorigenesis in several tumour types and animal tumour models^46^. Although *ras* mutations are rare in breast cancer, aberrant activation of the RAS protein and its downstream effectors is common and occurs as a result of amplification of *KRAS*^47^. Moreover, *ras* mutations are rarely found together with PI3K mutations in breast tumours^39^, again indicating engagement of overlapping signalling pathways during tumorigenesis.

There is an increasing number of observations hinting at the possible role of aberrant RAS signalling also in the emergence of innate and acquired resistance against ErbB2 targeting. For instance, modified SKBR3 cells overexpressing HRAS or harbouring the oncogenic allele *HRAS V12* displayed reduced susceptibility to lapatinib, in analogy to what was observed with activating *PIK3CA* mutations or the constitutively active form of AKT^48^. Moreover, *in vitro* and *in silico* evaluations have uncovered *ras* mutation as a dominant predictor of resistance to PI3K inhibitors in cancers of different origin including breast tumours overexpressing ErbB2 (refs 49, 50, 51). Thus, although the clinical relevance of the RAS-mediated resistance against ErbB2 blockade still needs to be proven, promising results from phase I studies of the RAS FT inhibitor lonafarnib, administered in combination with trastuzumab plus paclitaxel—showing a respectable objective response rate of 58% in treatment of advanced ErbB2-overexpressing breast cancer—or regimens combining AKT inhibitor MK2206 with trastuzumab, underscore the potential of therapeutic intervention at the RAS-PI3K-AKT axis in the treatment of ErbB2-addicted breast cancers^52^,^53^.

In the absence of external perturbations, the growth of ErbB2-dependent tumours occurs with operational AKT-ErbB3-negative feedback, that is, leading to lower ErbB3 levels, as a result of continued stimulation of AKT. Therefore, RAS inhibitor treatment, which interferes with the RAS-PI3K/AKT crosstalk, induces relief of this feedback and leads to upregulation of ErbB3, gradual re-phosphorylation of AKT and, consequently, to desensitization to RAS inhibitors, since then the relative flux through the ErbB3/PI3K/AKT pathway increases. However, if RAS inhibitors are combined with agents antagonizing the ErbB3 phosphorylation, such as trastuzumab, the outcome of RAS inhibition becomes substantially potentiated and comparable to the cytotoxic effects of RAS inhibitors observed for RAS-driven tumours.

As outlined above, the dephosphorylation of ErbB3 receptor, even if persisting for periods of days, is not sufficient to induce complete and sustained inhibition of AKT, explaining why all currently available anti-ErbB3 compounds failed to induce apoptosis as single agents^13^,^14^. Moreover, we and others showed that even the ErbB3 depletion by siRNA was not enough to elicit an apoptotic response in ErbB2-dependent tumours, although it potently inhibited proliferation of tumour cells^21^. Hence, the targeting of ErbB3 alone is not sufficient despite the indisputable role of ErbB3 in ErbB2-driven oncogenesis. The same accounts for prototypical ErbB2-targeting agents such as trastuzumab or pertuzumab, which only exert their effect on decoupling of ErbB3 from ErbB2, but leaving the phosphorylated ErbB2 unaffected (Fig. 7a). On the other hand, TKIs targeting solely ErbB2 usually suffer from the aforementioned reactivation of ErbB3, due to relief of the negative feedback with AKT, so that ErbB2/3 signalling is buffered against a nearly two-log inhibition of ErbB2 catalytic activity^43^.

The close mechanistic linkage between ErbB2 and ErbB3 thus entails the necessity to inhibit *both* ErbB2 and ErbB3 for an efficient targeting and silencing of ErbB2/3 signalling (Fig. 10c). This can be achieved by disassembly of all ErbB2-dependent complexes in a pan-ErbB approach, accompanied by inactivation of the receptor tyrosine kinases, as described in this work. By targeting two distinct epitopes of ErbB2 with appropriately designed biparatopic DARPins^31^ that completely disengage ErbB2 from the signalling network.

In conclusion, our study has untangled the mechanism underlying cancer cell-specific apoptotic response in ErbB2-addicted cells, revisited the individual roles of ErbB2 and ErbB3 receptors in tumour targeting and defined a new, as yet disregarded adaptive response operating via RAS-PI3K/AKT crosstalk, which leads to a desensitization towards current therapeutics. The new insights arising from this study can thus translate to improvement of current therapeutic modalities and aid in the rational design of a novel class of cell-specific extracellular receptor-targeting therapeutics with improved tumoricidal activity, low side effects and potential to avoid development of drug resistance. Nonetheless, an optimal targeting format of the biparatopic anti-ErbB2 DARPins^31^, with improved PK/PD profile while retaining the high pro-apoptotic activity, is still to be defined in ongoing *in vivo* studies. Furthermore, it will have to be ascertained in future preclinical and clinical experiments whether the induction of apoptosis in tumours during DARPin monotherapy is rapid and potent enough in order to eliminate cells that otherwise would acquire new cancerous mutations, bearing, for example, constitutively active PI3K, and to what degree cells from heterogeneous breast tumours, harbouring preexisting mutations, would have a selective growth advantage. We believe, however, that the structure- and mechanism-based engineering of ErbB2 ligands, which potently induce cell-specific apoptosis and diminish adaptive response, may provide a foothold for development of truly effective combination therapies to combat resistant tumours.

## Methods

### XTT cell proliferation assay

Cells were seeded in 96-well plates at a density of 2,000–10 000 cells per cm^2^ predetermined for each cell line. After 24 h, DARPins or mAbs were added in triplicates, and cells were incubated for another 96 h, unless indicated otherwise. In XTT assays (Cell proliferation kit II; Roche), A_450_ was measured and expressed as % untreated control.

### Clonogenic assay

BT474 cells were treated for 4 days with 100 nM of anti-ErbB2 agents, collected by trypsinization, washed 3 × with complete culture medium, serially diluted and seeded in fresh 6-well plates. Colonies were grown for 5 weeks, and the medium was refreshed twice per week. Colonies were fixed with glutaraldehyde, stained with crystal violet and the colony number was determined in triplicate for each single dilution.

### 3D growth assay

BT474 cells were seeded overnight at 1 × 10^4^ cells per cm^2^ on top of Matrigel (BD Biosciences). Cells were treated with 100 nM targeting agents in the absence or presence of 1 nM HRG, and the medium was refreshed every 3 days. Growth and morphogenesis of acini were analysed after 12 days by a modified XTT assay or by counting budding sites for 3 times 100 acini per treatment, respectively.

### Flow cytometry: cell cycle, TUNEL and annexin V assays

Cells were seeded at 5,000 to 10,000 cells per cm^2^ under standard conditions 24 h prior to treatment with 100 nM of anti-ErbB2 agents. After 3 days, cells were collected by trypsinization and fixed in 70% EtOH or in 4% paraformaldehyde for propidium iodide (20 μg ml^−1^; 2 Kunitz units per ml of RNAse) or TUNEL labelling (*In situ* cell death detection kit, Roche), respectively. The AnnexinV/PI assay was performed with non-fixed cells treated for 2 days (ApoDETECT Kit; Life Technologies). The cell cycle distribution and apoptosis rate were quantified with FlowJo software.

### Mouse xenograft studies

Eight-week-old female SCID beige mice were obtained from Charles River Laboratories and implanted with 0.025-mg, 90-day release, 17β-estradiol pellets (Innovative Research of America). After 2 days, 2 × 10^6^ BT474 cells were resuspended in 100 μl of PBS and 1:1 mixture with Matrigel was inoculated orthotopically into the mammary gland fat pad. Once tumours reached a volume of 200 mm^3^, 6–8 animals were selected and randomly assigned into four treatment cohorts with equal average tumour volumes. The DARPins were administered by i.v. injection three times per week in a total of nine doses of 20 mg kg^−1^. Tumour dimensions were serially measured every 2 days, and volumes calculated using the formula *V*=(*L* × *W*^2^)/2, where *V*=volume, *L*=length and *W*=width. For immunohistochemistry, animals were perfused and tumours were resected on day 14 after initiation of treatment (Supplementary Experimental Procedures). Bioimaging studies were performed after single i.p. injection of 1 mg kg^−1^ DARPin on an IVIS 100 instrument (Caliper Life Sciences), and biodistribution was quantified with Living Image software. All mice were maintained and handled under aseptic conditions. The studies were approved by the Cantonal Veterinary Office (Zurich, Switzerland). Housing and experimental procedures were in accordance with the Swiss animal protection law (Supplementary Experimental Procedures).

### Statistical analysis

All data are expressed as means±standard deviation (s.d.) unless indicated otherwise. The statistical analysis for two-group comparisons was performed in the following order: (i) Student's *t*-test for normally distributed samples with equal variance, (ii) Welch *t*-test for normally distributed samples with unequal variance (iii) Mann–Whitney Rank Sum Test for non-normally distributed samples. Next, we have included multiple sample comparisons versus control analysis by ANOVA followed by *post hoc* Dunnett's test. Significance was established at the *P*≤0.05; 0.01 and 0.001 level, as indicated in the figures.

## Additional information

**How to cite this article:** Tamaskovic, R. *et al*. Intermolecular biparatopic trapping of ErbB2 prevents compensatory activation of PI3K/AKT via RAS–p110 crosstalk. *Nat. Commun.* 7:11672 doi: 10.1038/ncomms11672 (2016).

## Supplementary Material

Supplementary Information Supplementary Figures 1-6, Supplementary Methods and Supplementary References

Supplementary Movie 1 Induction of Apoptosis by Bi-paratopic DARPins in Comparison to Trastuzumab Treatment. (S1-S2) BT474 cells were seeded 24 h before treatment in RPMI1640 containing 10 % FBS in a 12- well dish. Annexin V-Alexa488 (Life Technologies) was added to give a 1:50 final dilution and propidium iodide (2 g/l) was added to give a 1:2000 final dilution. Afterwards, cells were treated with 100 nM of trastuzumab. Phosphatidylserine flipping (green fluorescence, early apoptosis), loss of cell membrane integrity (red fluorescence, late apoptosis) and bright field images were recorded each 5 min for 48 h on a Lumascope 600 (etaluma) with the lowest possible LED power. Composite images were obtained by the manufacturer's software and videos were put together using the time lapse assembler.

Supplementary Movie 2 Induction of Apoptosis by Bi-paratopic DARPins in Comparison to Trastuzumab Treatment. BT474 cells were seeded 24 h before treatment in RPMI1640 containing 10 % FBS in a 12- well dish. Annexin V-Alexa488 (Life Technologies) was added to give a 1:50 final dilution and propidium iodide (2 g/l) was added to give a 1:2000 final dilution. Afterwards, cells were treated with 100 nM of 6L1G. Phosphatidylserine flipping (green fluorescence, early apoptosis), loss of cell membrane integrity (red fluorescence, late apoptosis) and bright field images were recorded each 5 min for 48 h on a Lumascope 600 (etaluma) with the lowest possible LED power. Composite images were obtained by the manufacturer's software and videos were put together using the time lapse assembler.
