# Supplementary material for: Intermolecular biparatopic trapping of ErbB2 prevents compensatory activation of PI3K/AKT via RAS–p110 crosstalk
Source: Nat Commun. 2016 Jun 3;7:11672. doi: 10.1038/ncomms11672 (PMC4895728; doi:10.1038/ncomms11672)
Supplement: Supplementary Information — Supplementary Figures 1-6, Supplementary Methods and Supplementary References [file ncomms11672-s1.pdf]

Supplementary Figure 1. Bispecific DARPins induce ErbB2/ErbB3 Dephosphorylation, while ErbB2 Receptor Remains Expressed on the Cell Surface

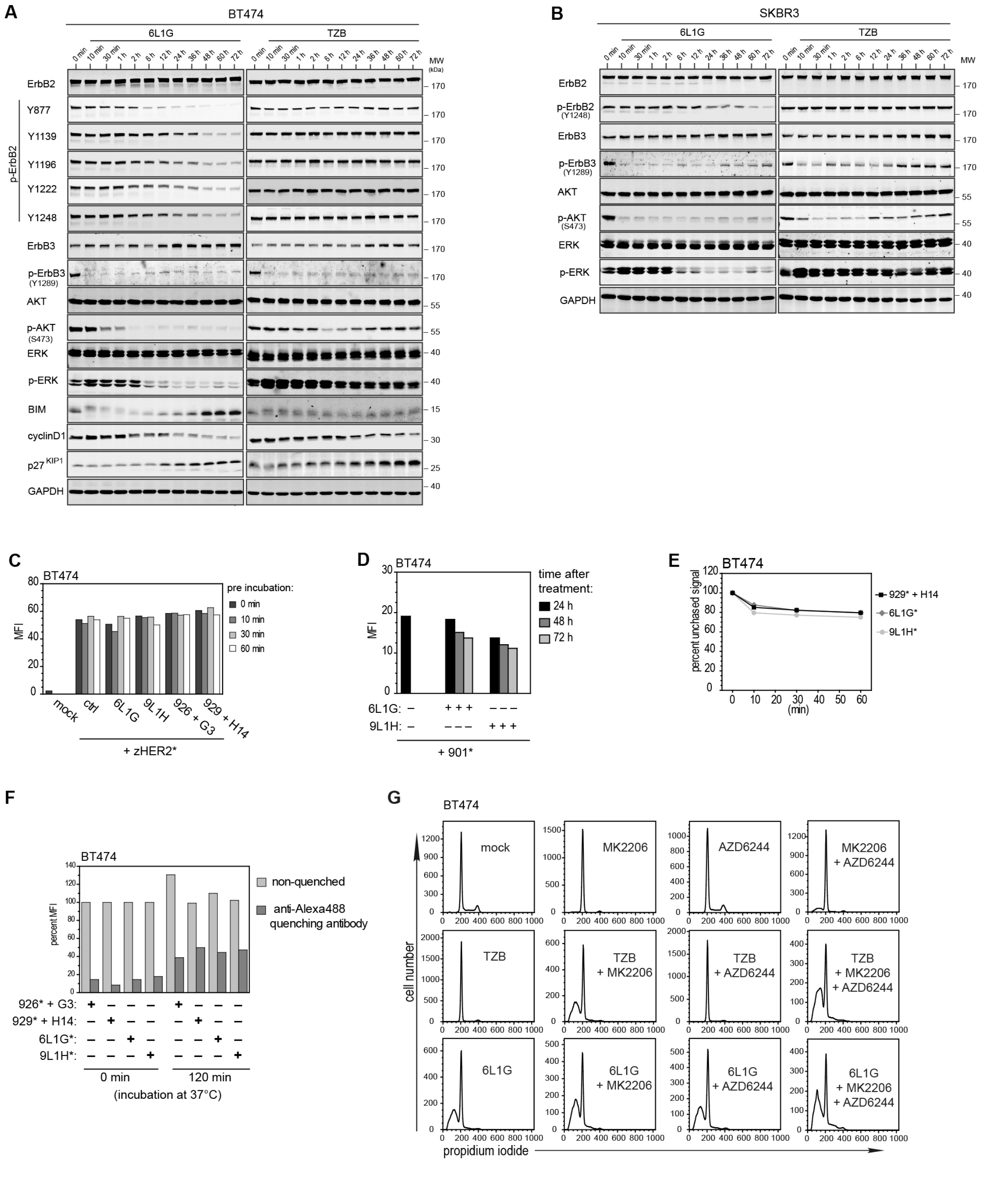

(A-B) Time-dependent western blot analyses using quantitative infrared detection (Licor Odyssey). Phosphorylation at indicated single Tyr sites of ErbB2, ErbB3, AKT, ERK (T202/Y204) together with expression of $p27^{KIP1}$, cyclinD1, BIM and GAPDH (control) were recorded in BT474 *(A)* and SKBR3 *(B)* cells after treatment with bispecific DARPin (6L1G) or trastuzumab (TZB) for indicated times. Shown are representative western blots of Fig. 1C-F.

(C-F) Active bispecific DARPins do not trigger increased ErbB2 internalization compared to inactive DARPins. Receptor internalization coupled to degradation can in principle attenuate oncogenic signaling by committing surface receptors for lysosomal degradation. We investigated whether these biparatopic DARPins influence the surface expression level or the internalization rate of the ErbB2 receptor. In *(C)* ErbB2 expression levels were measured by FACS using $Alexa_{488}$-conjugated anti-ErbB2 affibody (zHER2*[1], the asterisk indicating the label). zHER2* was bound to ErbB2 on the surface of BT474 cells, which were pre-treated with the indicated DARPins for indicated times. Note that zHER2* binds to ErbB2_ECD_III and does not compete with monovalent DARPins (926, 929, G3, H14) or bispecific counterparts for binding to ErbB2 receptor (data not shown). In *(D)* ErbB2 expression levels were measured by FACS using $Alexa_{488}$-conjugated monovalent DARPin (901*, the asterisk indicating the label). 901* was bound to the surface of BT474 cells after preincubation with indicated DARPins for indicated times. In *(E)*, a pulse-chase-assay was performed by FACS, detecting the quenching of mono- and bivalent DARPins directly conjugated with $Alexa_{488}$ (indicated by an asterisk) by an anti-$Alexa_{488}$ antibody. Receptor-bound DARPins are recycling back after internalization to the cell surface, remaining bound to ErbB2[2]. The indicated agents were preincubated for 2 h at 37 °C to allow for internalization, fluorescence of $Alexa_{488}$-conjugated DARPins was quenched by an anti-$Alexa_{488}$ antibody at 4°C, and afterwards, cells were incubated for indicated times in the presence of the quenching antibody at 37°C. Remaining fluorescence was measured by FACS. Active and inactive constructs recycle with the same rate. In *(F)*, an internalization assay performed by FACS is shown, detecting mono- and bivalent DARPins that were directly conjugated with $Alexa_{488}$ (indicated by an asterisk), incubated at 100 nM concentration for 2 h on BT474 cells. Afterwards, surface fluorescence was quenched by incubation with an anti-$Alexa_{488}$ antibody at 4°C and residual fluorescence was measured by FACS. Active and inactive constructs internalize with the same rate. Taken together, these findings imply that the bispecific anti-ErbB2 DARPins achieve their cytotoxic activity by inhibition of ErbB2 receptor phosphorylation rather than downregulation of ErbB2 expression. ErbB2 thus behaves differently to what we and others reported for binders targeting the EGF receptor[2,3].

(G) Sustained AKT inhibition by MK2206 is not sufficient to induce apoptosis, while combination of trastuzumab and MK2206 elicits apoptosis in presence of p-ERK. FACS analysis of cell cycle distribution of BT474 cells after 72 h treatment with the AKT inhibitor

MK2206 (1 µM), MEK1 inhibitor AZD6244 (2 µM), TZB (100 nM), 6L1G (100 nM) or their indicated combinations. MK2206 treatment induces a reduction of cells in S- and G2- phase and leads to accumulation in G1-phase. Treatment with AZD6244 did not change the cell cycle profile. Combination treatment of MK2206 and AZD6244 did induce the formation a small sub-G1 population, which however in comparison to DARPin 6L1G mono-treatment was negligible. In comparison, combination of MK2206 with TZB did strongly induce the formation of sub-G1 cells and therefore clearly shows strong signs of apoptosis. Further addition of AZD6244 did increase the population of cell with sub-G1 DNA content. However, combination of AZD6244 with TZB did not induce the formation of sub-G1 cells. DARPin 6L1G did show strong signs of apoptosis in all combinations with the small molecule inhibitors.

Supplementary Figure 2. Anti-Proliferative Activity Bi-paratopic and Monovalent DARPins

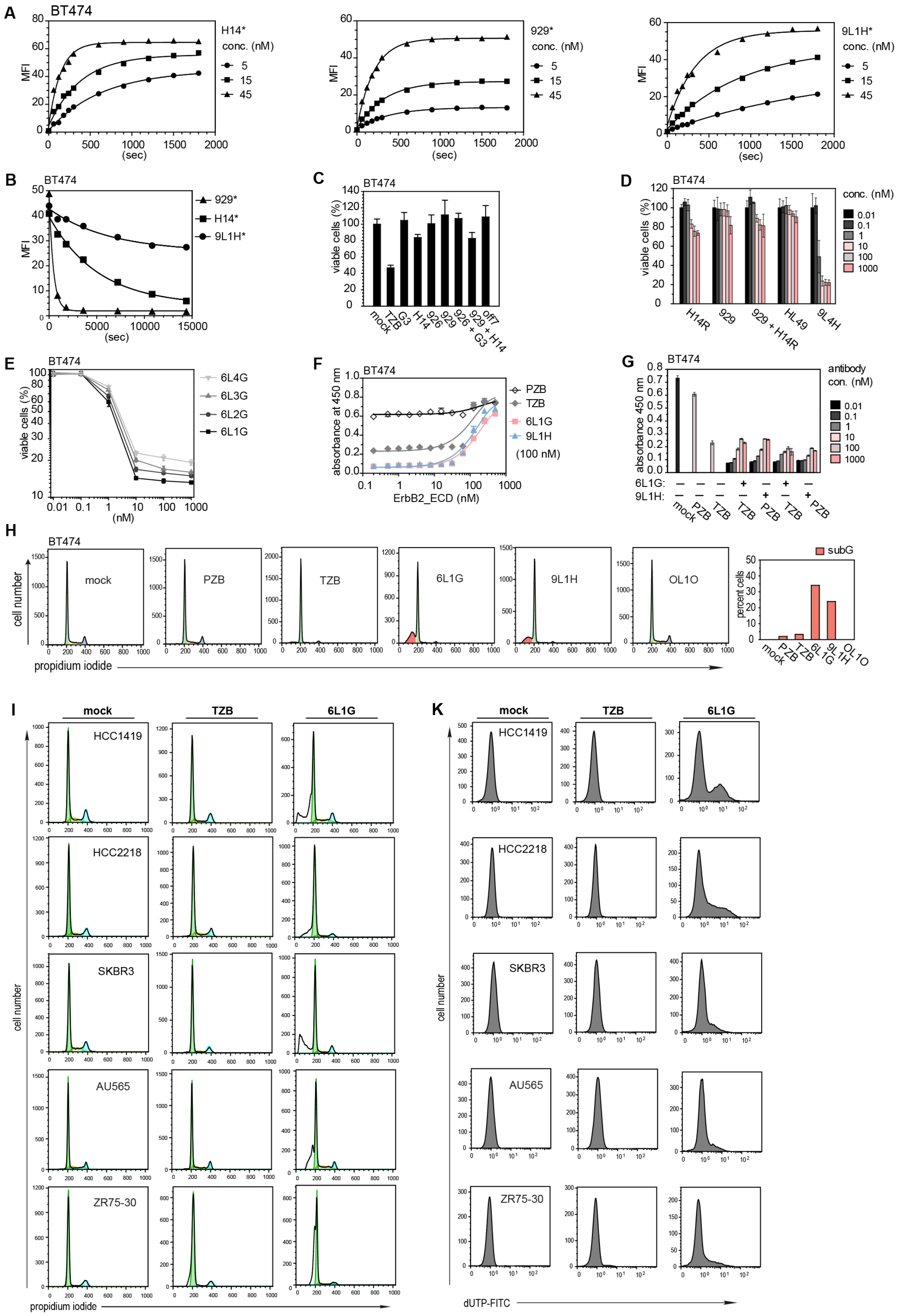

Cell Surface Binding, Combination Treatment with Antibodies, Cell Cycle and TUNEL Assay after Treatment with Bi-paratopic DARPins or their Monovalent Counterparts

(A-B) Association and dissociate rates of mono- and bivalent DARPins to ErbB2 receptor on the surface of BT474 cells. (*A*) Association rates were measured at different DARPin concentrations. Internalization was inhibited with azide prior to incubation with DARPin. $Alexa_{488}$-conjugated DARPins (indicated by an asterisk) were incubated for the indicated times with the indicated concentration and mean fluorescence intensity (MFI) was recorded by FACS measurements. Association rate constants of $2.0 \cdot 10^5$ M^{-1} s^{-1} for H14, $0.7 \cdot 10^5$ M^{-1} s^{-1} for DARPin 929, and $0.8 \cdot 10^5$ M^{-1} s^{-1} for 9L4H were obtained. (*B*) Dissociation rate of mono- and bivalent DARPins from ErbB2 receptor on the surface of BT474 cells. Internalization was inhibited with azide prior to incubation with DARPin. $Alexa_{488}$-conjugated DARPins were bound to saturation, subsequently DARPins were competed for the indicated times with an excess of their respective non-labeled DARPin counterparts and MFI values were recorded by FACS measurements. Dissociation rate constants of $1.8 \cdot 10^{-4}$ s^{-1} for DARPin H14, $2.2 \cdot 10^{-3}$ s^{-1} for DARPin 929, and $4.0 \cdot 10^{-5}$ s^{-1} for DARPin 9L1H were obtained. From *(A)* and *(B)*, functional affinities of K_D = 1 nM can be calculated for DARPin H14, 33.5 nM for 929, and 0.5 nM for 9L1H on the surface of BT474 cells. From the markedly lower dissociation rate constants of the bispecific DARPins on BT474 cells, compared to their respective monovalent DARPin constituents, we conclude that in the bispecific anti-ErbB2 DARPins both DARPin domains are engaged in binding.

(C-E) Anti-proliferative activity of different constructs. (*C*) XTT assays detecting anti-proliferative activity of monovalent DARPins (100 nM) and their combination (each 100 nM) on BT474 cells after 96 h treatment. Trastuzumab (TZB) and all DARPins bind to ErbB2, except Off7, which serves a negative control. (*D*) XTT assay demonstrating the necessity to link the DARPins to a particular bispecific format. BT474 cells were measured after 96 h treatment. The ErbB2_ECD_IV binding DARPin H14 displays a reduction of 20 % viability, the ErbB2_ECD_I binding DARPin 929 shows no anti-proliferative activity and their combination shows anti-proliferative activity similar to H14 treatment alone. The anti-proliferative activity of the bispecific DARPins is determined by the orientation of the monovalent DARPins within the bispecific constructs. The bispecific DARPin exist in an inactive orientation (HL49) and an active orientation (9L4H). The active orientation shows strong

anti-proliferative activity that exceeds simple additive effects by far. (*E*) XTT assay measuring the effect of the flexible linker length within the active bispecific DARPin on BT474 cells after 96 h treatment. Reducing the linker length from L4 = $(GGGGS)_4$ to L1 = (GGGGS) increases the maximal anti-proliferative activity.

(F) XTT assay showing the specificity of the anti-proliferative activity of bi-paratopic DARPins (6L1G and 9L1H) and the antibodies trastuzumab (TZB) and pertuzumab (PZB) by competition with soluble full-length ErbB2_ECD on BT474 cells after 96 h of treatment. Anti-ErbB2 agents (each 100 nM) were preincubated with increasing concentrations of ErbB2_ECD and given to the cancer cells. Increasing concentrations of ErbB2_ECD reduced the anti-proliferative activity of each anti-ErbB2 agents in a concentration-dependent manner to completion.

(G) XTT assay showing the effect of combining bi-paratopic DARPins at a constant concentration (100 nM) with increasing concentrations of either trastuzumab (TZB) or pertuzumab (PZB) on the anti-proliferative activity on BT474 cells after 96 h of treatment. With increasing concentration of either antibody, the strong anti-proliferative activity of bi-paratopic DARPins is markedly reduced. Note that H14 competes with TZB for binding to ErbB2_ECD_IV, while G3 does not (data not shown). None of the monovalent DARPins (926, 929, H14, G3) competes with PZB for binding to ErbB2_ECD (data no shown). Nonetheless, anti-proliferative activity of both bispecific DARPins is similarly reduced by both antibodies, respectively.

(H) FACS analysis of cell cycle distribution of BT474 cells after 72 h treatment with indicated agents, each at 100 nM concentration. Depicted propidium iodide (PI)-staining histograms were gated for single cells and quantified with the Dean-Jett-Fox algorithm. Cells with sub-G1 DNA content (*red*) represent apoptotic cell populations with marked degradation of genomic DNA.

(I) Cell cycle distribution analysis of ErbB2-overexpressing cancer cells after 72 h treatment with the indicated agents, each at 100 nM concentration. Ethanol-fixed cells were stained with propidium iodide (20 µg/ml) and analyzed by FACS. Depicted PI-staining histograms show cell cycle profiles of gated single cells and populations are assigned with the Dean-Jett-Fox algorithm.

(K) Cell death detection assays of ErbB2-overexpressing cancer cells treated for 72 h with indicated agents at 100 nM. Presented histograms depict the recorded TUNEL profiles of non-apoptotic (*left peak,* centered on 10^0*)* and apoptotic cells (*right peak,* with a maximum at up to 10^2)*.* After treatment, cells were PFA-fixed and Triton-permeabilized, stained with terminal deoxynucleotidyl transferase (TdT) using dUTP-fluorescein as substrate and subsequently analyzed by FACS.

Supplementary Figure 3. Anti-Proliferative Activity of PEGylated DARPins

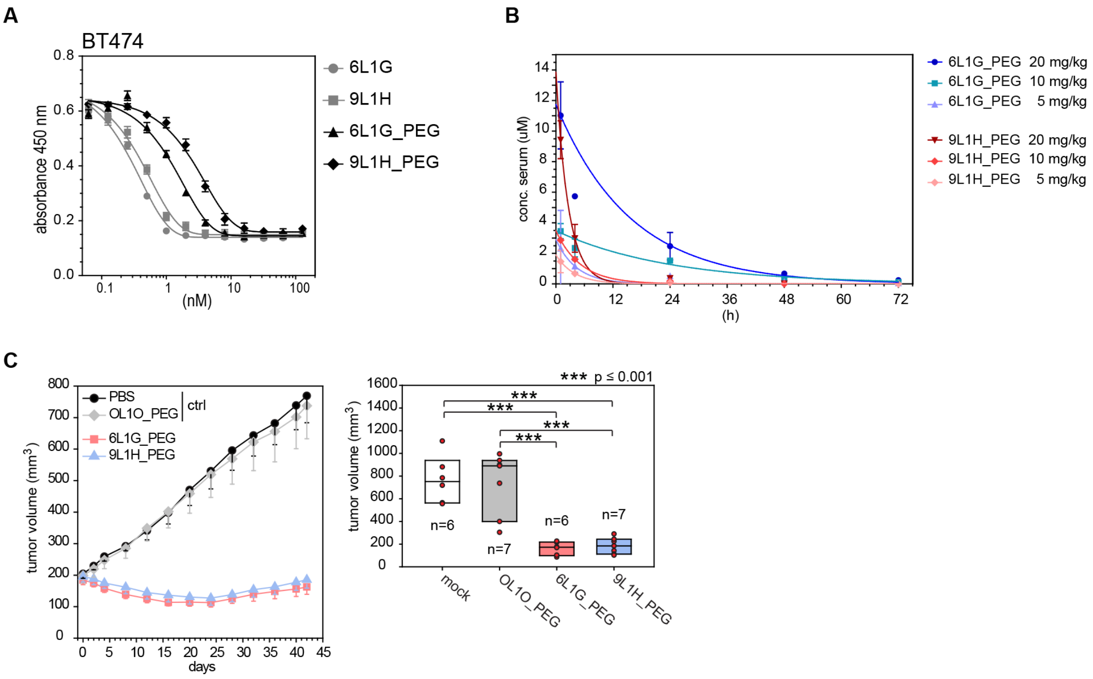

(A) XTT assay showing the effect of PEGylation on both bi-paratopic DARPins. The anti-proliferative activity was measured on BT474 cells after 96 h of treatment. The maximal anti-proliferative activity was not affected by N-terminal PEGylation, while dose effectiveness was reduced. IC_{50} values of non-conjugated DARPins increased from the picomolar range after PEGylation to low nanomolar concentrations.

(B) Quantification of PEGylated DARPins in serum of SCID beige mice after a single injection. PEGylated DARPins were injected intravenously at time 0 at the indicated concentration, blood samples were analyzed after indicated time and concentrations were determined by a reference titration of the same DARPin in a sandwich ELISA. The *in vivo* half-lives of the targeted PEGylated constructs were determined as 21.2 h (AUC 15.3 µmol/l·h) for 6L1G_PEG and 7.0 h (AUC 5.8 µmol/l·h) for 9L1H_PEG, being at least two orders of magnitude higher than the underivatized counterparts[4].

(C) Tumor growth inhibition by PEGylated DARPins in SCID beige mice with pre-established BT474 xenografts (same as in Fig. 3B). Tumor size distribution of individual treatment groups after 44 days is plotted below (**P* values were calculated using two-sided Students *t*-test).

Supplementary Figure 4. Individual Contribution of ErbB2 and ErbB3 Receptor to Survival of ErbB2 Overexpressing Cancer Cells

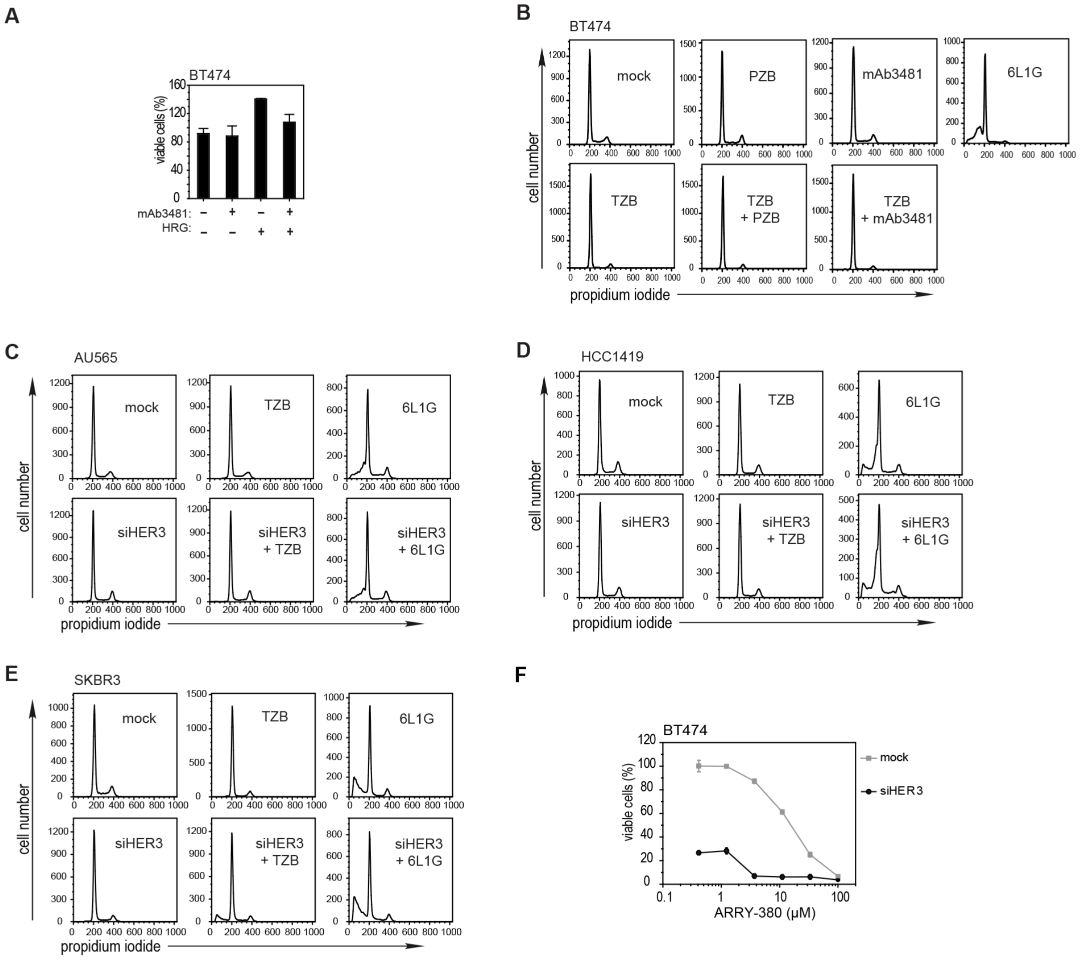

(A) XTT assay with BT474 cells after 96 h treatment with the anti-ErbB3 antibody mAb3481 (50 nM) in the absence or presence of HRG (1 nM) shows that the anti-ErbB3 antibody efficiently reduces HRG-induced growth, while it did not markedly effect cell growth in the absence of HRG stimulation.

(B) FACS analysis of cell cycle distribution of BT474 cells treated with a combination of pertuzumab (100 nM) or mAb3481 (50 nM) and trastuzumab (100 nM). Cell cycle profiles indicate that addition of either antibody to trastuzumab did not trigger formation of cells with sub-G_1 DNA content, in contrast to DARPin 6L1G which serves here as positive control.

(C-E) Inhibition of cell cycle progression and formation of sub-G_1 population in AU565 (*D*), HCC1419 (*E*) and SKBR3 (*F*) cells 72 h after ErbB3 knockdown by siRNA (siHER3) alone or in combination with TZB (100 nM) or DARPin 6L1G (100 nM) treatment. Knock down of ErbB3 did not induce formation of a marked sub-G_1 apoptotic cell population and predominantly

reduced the amount of cells in S- and G_2-phase. Combination treatment of siHER3 and TZB did not markedly add to the effects of single siHER3 treatment in these cells, while DARPin 6L1G consistently induced formation of apoptotic cells in all cell lines.

(F) XTT assay with BT474 cells after 96 h treatment with a titration of the selective ErbB2 kinase inhibitor ARRY-380 alone and in combination with ErbB3 knock-down by siHER3. The IC_{50} of ARRY-380 is shifted towards the low µM range in the absence of ErbB3 receptor expression, indicating synergy for the inhibition of ErbB2 together with ErbB3.

Supplementary Figure 5. PI3K Dependency and RAS Bypass of ErbB3 for Reactivation of PI3K/AKT Pathway

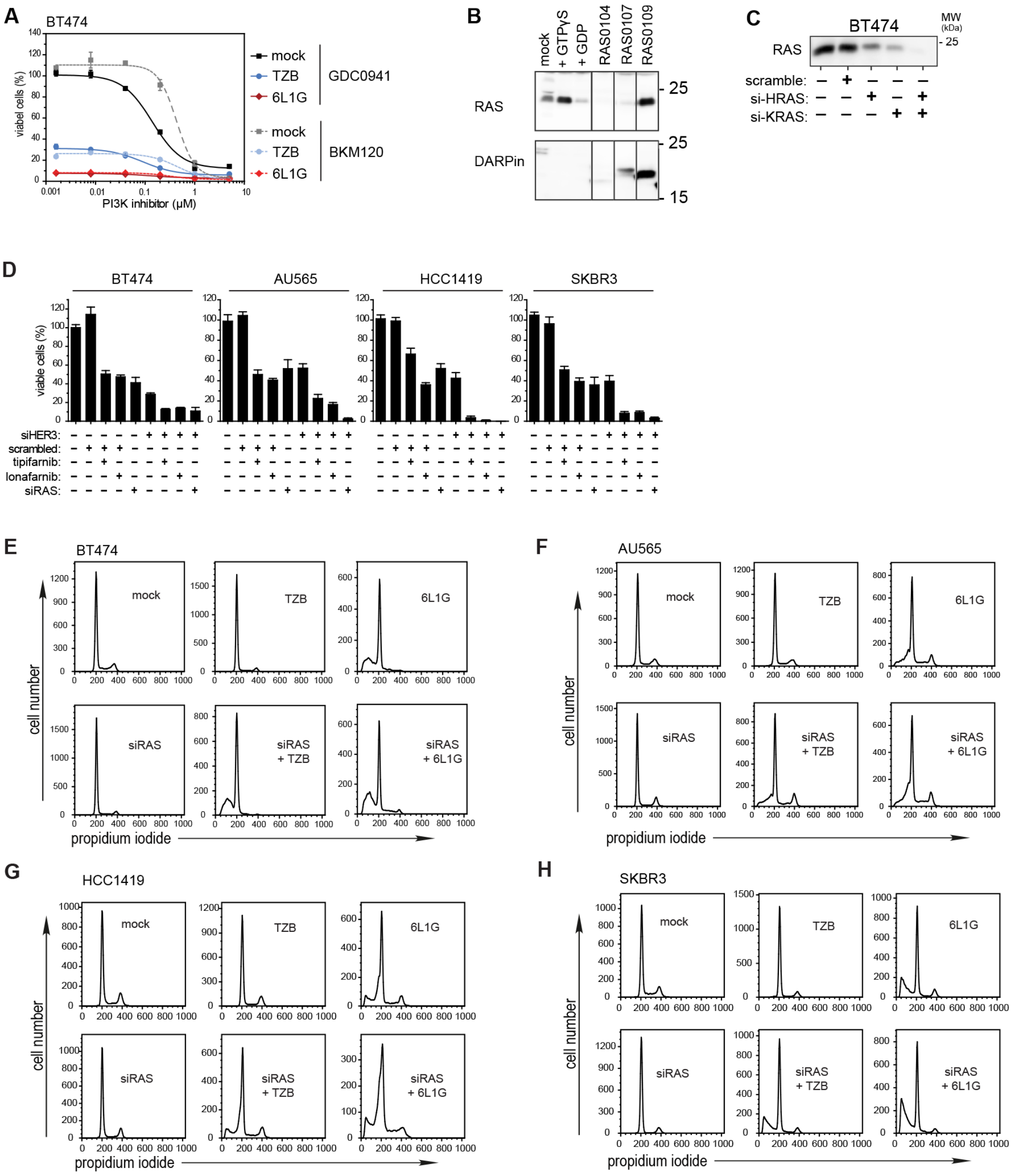

(A) XTT assay of BT474 cells after 96 h of treatment with a titration of the PI3K inhibitors GDC-0941 or BKM-120 alone and in combination with either trastuzumab (100 nM) or DARPin 6L1G (100 nM). BT474 cells show high sensitivity to both PI3K inhibitors which is similar to cell lines that carry a PI3K activating mutation (data not shown). Note that combination of TZB with either PI3K inhibitor becomes similarly active as a DARPin 6L1G treatment by itself in BT474 cells.

(B) Anti-Ras DARPin RAS104 and RAS107 competed with RBD binding, while DARPin RAS109 did not compete with RAS RBD binding and was co-immunoprecipiated. Cell extracts were

stimulated with GTPγS and co-incubated with 10 µM of either DARPin RAS104, RAS107 or RAS0109. RAS was immunoprecipitated by RBD-GST on glutathione beads and detected by anti- pan-RAS on western blot, while co-precipitating DARPin was detected in an anti-DARPin blot.

(C) Western blot analysis of pan-RAS expression 72 h after siRNA knock-down with either of siHRAS (50 nM), siKRAS (50 nM) or both.

(D) XTT assay of BT474, AU565, HCC1419 and SKBR3 cells with and without ErbB3 knock-down after 96 h incubation. Combination treatment of siHER3 with either lonafarnib (5 µM) or tipifarnib (5 µM) strongly reduced the cell proliferation with respect to the single ErbB3 knock-down. Similarly, the combination of the HRAS/KRAS and ErbB3 knock-down more potently blocked the cell growth of BT474 cells than the single ErbB3 knock-down.

(E-H) FACS analysis of cell cycle distribution of BT474 (*E*), AU565 (*F*), HCC1419 (*G*) and SKBR3 (*H*) cells after 72 h of treatment with HRAS/KRAS knock-down (siRAS). Cancer cells were treated with the siRAS cocktail and did not show marked formation of cell sub-G_1 DNA content. Combination treatment with either trastuzumab (100 nM) or DARPin 6L1G (100 nM) induced a strong formation of a sub-G_1 population.

Supplementary Figure 6. ErbB2 autophosphorylation does not require Src, Src inhibition does not block p-AKT and dominant active Src does not prevent apoptosis after TZB/TIP or 6L1G

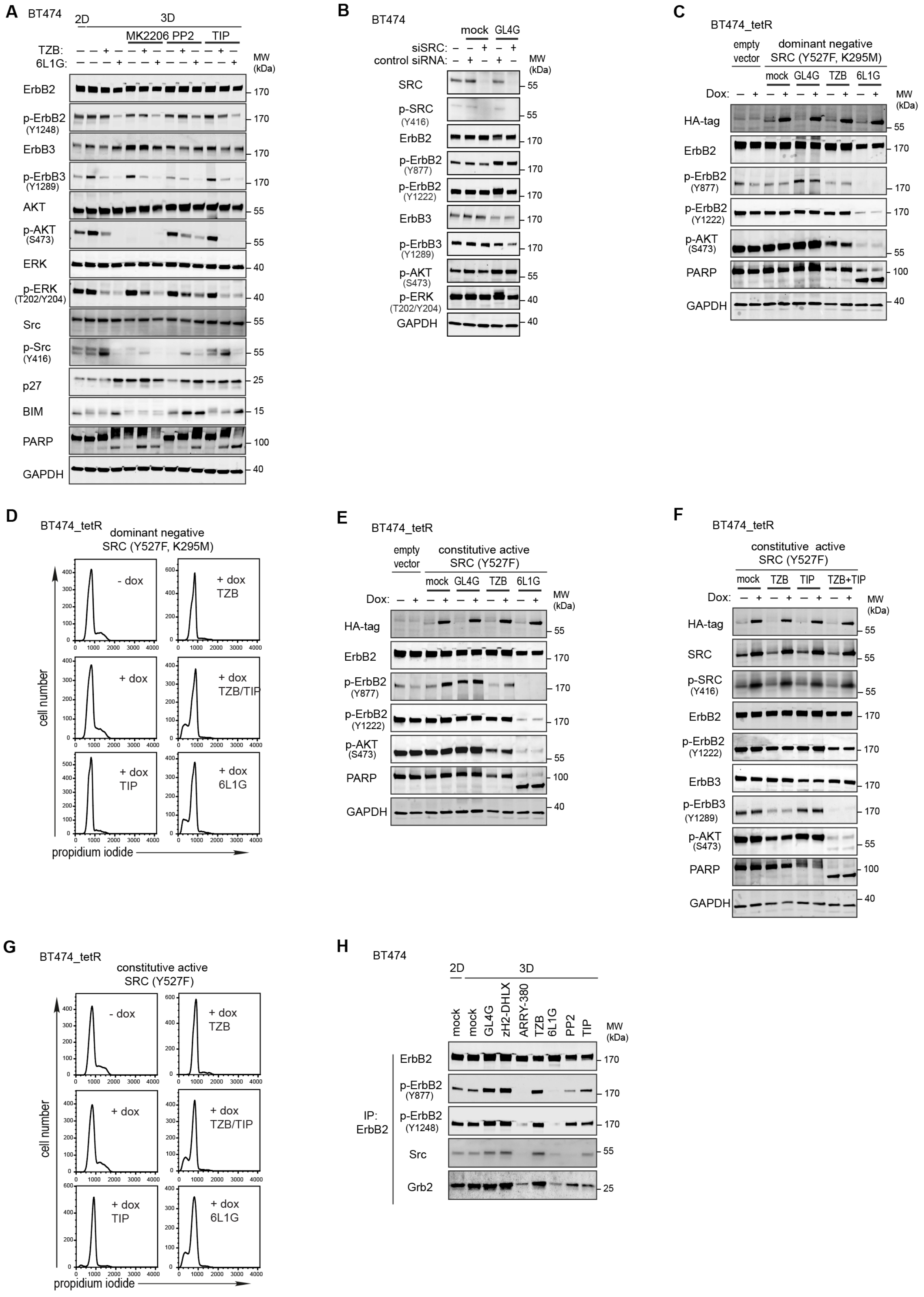

(A) BT474 cells were seeded 48 h prior treatment in poly-HEMA-coated dishes to induce 3D growth. Cells were treated with either with 1 µM AKT inhibitor (MK2206), 10 µM Src inhibitor (PP2), 12.5 µM ErbB2 inhibitor (ARRY-380) or 5 µM RAS farnesylation inhibitor (tipifarnib/TIP). Furthermore, 100 nM of trastuzumab or 6L1G was added. Cells were analyzed 72 h after treatment. Combination of either AKT or RAS inhibitor with TZB induced complete AKT dephosphorylation and led to induction of PARP cleavage. Combination of PP2 with TZB did not induce complete AKT dephosphorylation and did not show marked PARP cleavage.

(B) ErbB2 autophosphorylation does not require Src in BT474 cells. Analysis was done 72 h after knockdown of Src by siRNA in combination with 50 nM of the ErbB2-activating molecule GL4G. Src knockdown was not sufficient to block formation of p-ErbB2, p-ErbB3 and p-AKT. Stimulation of ErbB2 homodimers by GL4G, in the absence of Src expression, was sufficient to stimulate ErbB2 phosphorylation at Y877.

(C-G) Expression of HA-tagged human dominant-negative Src (Y527F, K295M) (*C,D*) or constitutively active Src (Y529F) (*E,F,G*) was induced in stable BT474_TetR cells with 1 µg/ml doxycycline. Cells were treated for 72 h with either 50 nM GL4G, 100 nM trastuzumab, 100 nM 6L1G, 5 µM tipifarnib (TIP) or the indicated combination, and analyzed by western blot or FACS. Induced expression of dominant-negative Src did not block p-ErbB2 or p-AKT (*C*) and did not induce apoptosis (*D*). Induced expression of dominant negative Src did not block the p-AKT rebound after TZB treatment (*C*) and also did not induce a marked subG1 population (*D*). Expression of constitutively active Src (Y527F) lead to upregulation of ErbB2 Y877 phosphorylation independent of treatment with TZB (*E*). However, 6L1G treatment blocked p-ErbB2 and induced PARP cleavage in the presence of induced expression of constitutively active Src (*E*). Combination treatment of TZB/TIP did block p-AKT rebound (F) and did induce a subG1 population (*G*) in the presence of constitutively active Src. Thus, induced expression of constitutively active Src (Y527F) does not prevent the induction of apoptosis after sustained inhibition of p-AKT and p-HER3 by TZB/TIP or 6L1G treatment (*E-F*).

(H) BT474 cell were treated as in (*A*), afterwards ErbB2 was immunoprecipitated with ErbB2_ECD binding agent 901-L4-zHER2 and analyzed by western blots for Co-IP of Src and Grb2. ARRY-380 and 6L1G blocked ErbB2 phosphorylation at Y877 and Y1248, and both

agents blocked Co-IP of Src and Grb2. PP2 blocked Co-IP of ErbB2-Src and partially ErbB2 phosphorylation at Y877 but not Co-IP of ErbB2-Grb2.

Supplementary Methods

Reagents

Antibodies against the following targets were used, and their manufacturers and catalogue numbers are listed: phospho-ErbB3 (Y1289) (#4791), phospho-ErbB2 (Y877) (#2241), phospho-ErbB2 (Y1196) (#6942), phospho-ErbB2 (Y1222) (#2249), phospho-ERK (T202/Y204) (#4370), ERK (#4695), phospho-AKT (S473) (#4060), pan-AKT (#4691), PARP (#9542), p110α (PI3K) (#4249), pan RAS (#8832), Grb2 (in Fig. 9E) (#3972), cyclinD1 (#2926), caspase 3 (#9662), BIM (#2933), EGFR (#4267), from Cell Signaling; Phospho-ErbB2 (Y1139) (ab53290) from Abcam; ErbB2 mouse (3B5) (OP15), phospho-ErbB2 (1248) (06-229) from Calbiochem Merck Millipore; GAPDH (sc-365062), Grb2 (sc-255) (in Fig. 1A), ErbB3 rabbit mAb (C-17) (sc-285) from Santa Cruz; ErbB2 (4B5) from Ventana; anti-mouse IgG IRDye800 conjugate from Rockland (610-732-124); anti-$Alexa_{488}$ quenching antibody (A-11094), anti-rabbit IgG $Alexa_{680}$ (A-21109) from Invitrogen; anti-phospho-Tyr (pY20) (P4110) and anti-hemagglutinin tag (HA-Tag) (SAB4300603) from Sigma; $p27^{KIP1}$ (610241) from BD Transduction Laboratories. The anti-DARPin polyclonal rabbit serum was obtained from Eurogentech. Primary detection antibodies were used at 1:1000 and secondary antibodies at 1:10000 dilution in 5 ml PBS or TRIS based blocking buffer solution. Anti-ErbB2 and anti-phospho-ErbB2 antibodies were used at 1:10000 and anti-ErbB3 antibodies were used at 1:200 dilution. PageRuler (SM0671) from Fermentas was used to determine molecular weight on 4-20% Tris-Glycine SDS-PAGE from BioRad and western blot. Trastuzumab (Herceptin®) was obtained from Kantonsapotheke Zürich. Pertuzumab (Omnitarg, Perjeta®) was a kind gift from Genentech Roche. The anti-ErbB3 antibody mAb3481 was obtained from R&D Systems. The affibody zHER2 was expressed in *E. coli* XL1 and purified as described below for DARPins. Recombinant human epidermal growth factor (hEGF) (6.22 kDa) was obtained from Jena Bioscience. Recombinant human heregulin-β1 (HRG, NRG1) (26.9 kDa) was obtained from R&D systems. RAS inhibitors tipifarnib and lonafarnib, EGFR/ErbB2 inhibitor lapatinib (lap), selective ErbB2 inhibitor ARRY-380 (ARRY), PI3K inhibitor GDC0941 and BKM-120, AKT inhibitor MK2206 and GDC0068, Src inhibitor PP2 and MEK inhibitor AZD6244 were obtained from Selleckchem. The siRNA against ErbB3 (SignalSilence® HER3/ErbB3 siRNAII) and siRNA against Grb2 (SignalSilence® GRB2 siRNA I) were obtained from Cell Signaling. Validated

siRNA's against HRAS (s806), KRAS (120703) and Src (s13414) were obtained from Ambion (Life Technologies, Thermofisher Scientific). The negative control siRNA (med GC) was obtained from Ambion (Life Technologies, Thermofisher Scientific). TNF-related apoptosis-inducing ligand (TRAIL) was obtained from Dr. Robbert H. Cool, University of Groningen, Netherlands. ErbB2_ECD was obtained from EMP Genetech (Ingolstadt, Germany) from expression in HEK cells.

Cell lines

The human mammary ductal carcinoma line BT474 was obtained from the American Type Culture Collection (ATCC; www.atcc.org) (ATCC HTB-20), as was the human mammary adenocarcinoma line SKBR3 (ATCC HTB-30), the human mammary adenocarcinoma line AU565 (ATCC CRL-2351), and the human mammary ductal carcinoma line ZR-75-30 (ATCC CRL-1504). The human mammary ductal carcinoma lines HCC1419 (ATCC CRL-2326) and HCC2218 (ATCC CRL-2343) were obtained from Dr. Nancy Hynes, Friedrich Miescher Institut, Basel. Human mammary epithelial cells HMEC were obtained from RUWAG (CC-2551), and human cardiac myocytes (HCM) from Innoprot (P10451-F).

Protease and Phosphatase Inhibitors

The following protease inhibitors were used at the indicated final concentrations: 1 mM pefabloc/AEBSF (Meck), 0.02 mM leupeptin (Serva), 0.01 mM pepstatin (Serva) and 0.02 mM marimastat (Calbiochem). The following phosphatase inhibitors were obtained from Sigma Aldrich and used at the indicated final concentrations: 1 mM sodium orthovanadate, 1 mM sodium metavanadate, 10 mM sodium molybdate, 50 mM sodium β-glycerol phosphate, 50 mM sodium fluoride.

Construction and Cloning of Bispecific and Biparatopic DARPins

In this study we used designed ankyrin repeat proteins[5,6], to construct potent bispecific agents. DARPins had been selected by phage display [7] which bind to domain I of ErbB2[8]; DARPin 926 was originally termed 9_26 (or 9.26), and DARPin 929 was termed 9_29 (or 9.29)[7]. Using ribosome display, DARPins had been selected and affinity-matured which bind

to domain IV, and DARPin G3 was originally termed H10-2-G3[9,10], while another domain IV binding DARPin, H14 (originally termed H_14), has been identified by phage display[7] and was derivatized here further with a C31R Mutation (H14R).

X-ray crystallography[8] has shown that the domain I-binding DARPins 929 and 926 recognize the same epitope, while the epitopes of G3 and H14 on domain IV overlap, but are not identical: both DARPins compete with each other, but only H14 competes with trastuzumab.

While all 4 bispecific combinations of N-terminal domain I and C-terminal domain IV binders lead to highly active molecules, we concentrate here on two of them, 926-Linker-G3 (abbreviated 6L1G) and 929-Linker-H14 (abbreviated 9L1H), where the linker length (L1) indicates a length of one GGGGS unit. To make these bispecific constructs, the DARPin ORFs were digested with BamHI and HindIII (New England Biolabs) and ligated into compatible expression vectors based on pQiB containing $(Gly_4Ser)_{1-4}$ linkers (abbreviated as L1, L2,L3 and L4), indicating the number of Gly_4Ser units.

ErbB2 activating homo-bivalent DARPin G3-L4-G3 (GL4G) and affibody zHER2-L4-DHLX (zH2-DHLX) were constructed in the pQiBi-22- vector. The disulfide linked leucine zipper domain (DHLX) (Kubetzko et al. 2006) was adapted to the following amino acid sequence: RSPSGELEELLKHLKELLKGPRKGEL**C**ELLKHLKEL**C**KGSPKLN. Note that the affibody DHLX domain fusions can be expressed in the cytoplasm of *E.Coli* XL1.

Expression and Purification

DARPin expression and purification has been described in detail previously[4]. Briefly, DARPins were expressed in pQE30-derived vectors in *E. coli* strains XL1 blue or BL21. DARPins were purified via their $MRGS(H)_6$ tag on Ni-NTA (Qiagen) bench-top columns, and endotoxin was removed by excessive washing with an additional 80 column volumes of PBS containing 0.1 % Triton-X115. DARPins were further purified by ion exchange chromatography on MonoQ columns (GE Healthcare) and finally size-exclusion chromatography on a Superdex 200 column (GE Healthcare) on an Aekta Explorer FPLC system (GE Healthcare). Endotoxin levels were determined by a LAL chromogenic endotoxin quantification kit (CRL) according to the manufacturer's protocol and protein samples displayed a maximum of 10 EU/ml.

Western Blots

Cells were seeded at a density of 5,000 to 10,000 cells/cm^2 24 h prior to treatment in RPMI1640 medium containing 10 % FCS. Cells were treated with 100 nM of anti-ErbB2 agents for the indicated times. Afterwards, cells were washed twice with cold PBS and scraped in PBS containing all inhibitors (see above). Note that apoptotic cells easily detach in PBS washing steps and were excluded from receptor signaling analysis. Cells were centrifuged at 300 rpm and washed once in cold PBS-containing inhibitors. Cells were lysed for 30 min at 4 °C in cold PBS containing 1 % Triton X-100 and inhibitors. Lysates were cleared by centrifugation at 20,000 *g* for 10 min and protein concentrations were determined by BCA assays (Pierce). Samples were adjusted to 2 mg/ml in reducing LDS sample buffer (Invitrogen) containing 0.05 % β-mercaptoethanol and boiled for 3 min. Samples were subjected to 10 % SDS-PAGE and blotted onto a PVDF-FL membrane (Millipore) using the wet blot protocol from Biorad. The membrane was blocked with PBS containing 1x casein blocking buffer (Sigma) and primary antibodies (see above) were incubated in PBS casein buffer containing 0.1 % Tween-20 overnight at 4°C. Membranes were washed three times in PBS containing 0.1% Tween-20 and fluorescence-labeled secondary antibodies (see above) were incubated for 2 h at room temperature. Membranes were washed 3 times in PBS 0.1% Tween-20 and fluorescence was detected on an Odyssey imaging system (LI-COR).

Immunoprecipitation of ErbB2 for Total Tyr Phosphorylation Analysis by Western Blot

BT474 cells were treated and cell lysates were obtained as described above. The ErbB2 receptor was immunoprecipitated using mouse anti-C terminal antibody 3B5 from (Merck Millipore), which was coupled to Biosupport Ultra Link beads (Pierce) overnight in 25 mM HEPES pH 8. ErbB2 receptor was immunoprecipitated from 1 mg/ml BT474 cell extracts overnight at 4 °C. Beads were washed three times with cold PBS containing 1 % Triton X-100 and inhibitors. ErbB2 receptor was eluted from beads by heating up to 80 °C for 5 min in reducing SDS sample buffer. Western blots were performed as described above.

Fluorescence Labeling and PEGylation of DARPins for In Vivo Experiments

$Alexa_{488}$ C_5 maleimide, $Alexa_{488}$ NHS ester, $Alexa_{680}$ NHS ester (Invitrogen) and IR_{800} NHS ester (LICOR) were coupled to proteins as described previously[11]. Branched PEG-40 maleimide (NOF Sunbright) was coupled to DARPins as described previously[11]. The PEGylated proteins were purified as described previously[4]. For following receptor internalization and/or recycling, the affibody zHER2 and the DARPin 901 were conjugated with $Alexa_{488}$ NHS ester as described previously[11] . The following DARPins, 926, 929, H14, G3, 6L1G, 9L1H, 9L4H were conjugated with $Alexa_{488}$ C_5 maleimide at a cysteine reside located between the $MRGSHis_6$-tag and the DARPin[11].

Crosslinking ErbB2-ErbB3 and ErbB2-EGFR Heterodimers with DTSSP and Co-immunoprecipitation

Cells were seeded at a density of 2.5 x 10^4 (BT474) or 5 x 10^4 (SKBR3) cells/cm^2 16 h before treatment in RPMI1640 medium containing 10 % FCS. Cells were treated for 2 h with 100 nM of anti-ErbB2 agents. Afterwards, cells were stimulated by adding 1 nM of heregulin β-1 or 2 nM of epidermal growth factor for 15 min. Subsequently, cells were placed on ice and washed 2 times with cold 25 mM HEPES buffer, pH 7.5, 150 mM sodium chloride and scraped gently in HEPES buffer. Cells were incubated with in HEPES buffer containing 1 mM 3,3'-dithiobis[sulfosuccinimidylpropionate] (DTSSP) for 1 h at 4 °C on a rocker. Afterwards, cells were washed twice with 50 mM Tris buffer pH 7.5, gently centrifuged at 300 *g* for 2 min in between washes. Cells were lysed using PBS buffer containing 1 % Triton X-100 and inhibitors at 4°C for 30 min and lysates were cleared by centrifugation at 20,000 *g* for 10 min at 4°C. Extracts (500 µl) containing 1 mg/ml protein, as determined by BCA assay (Pierce), were used for immunoprecipitation of ErbB2. Ultralink Biosupport beads (Pierce) were used that had been previously coupled with antibody recognizing the C-terminus of ErbB2 (Millipore) and immunoprecipitation was performed overnight at 4°C. Beads were washed 3 times with PBS containing all inhibitors and eluted by boiling in reducing SDS loading buffer for 5 min at 80 °C. Samples were subjected to 10 % SDS PAGE and western blot (see above).

Association and Dissociation Binding Analysis of Monovalent and Bivalent DARPins by FACS

The association and dissociation binding analysis to the surface of cancer cells was measured as described previously in detail[11]. Internalization was inhibited by incubation of cancer cells with sodium azide prior to binding analysis.

ErbB2 Receptor Cell Surface Expression Analysis by FACS

BT474 cells were seeded 24 h before treatment. Cells were incubated with $Alexa_{488}$-conjugated DARPin constructs for the indicated times. Cells were washed twice with PBS, collected by Accutase (PAA) treatment, washed once with culture medium and afterwards 2 times with cold PBS. Cells were incubated with 100 nM of either zHER2-$Alexa_{488}$ or 901-$Alexa_{488}$ for 1 h at 4°C. Afterwards, cells were washed 3 times with cold PBS, and mean fluorescence intensities were recorded on flow cytometer.

ErbB2 Receptor Internalization and Recycling Analysis by FACS

The internalization and recycling assay was described for EGFR previously[2,12] and carried out accordingly. The anti-ErbB2 bispecific DARPins presented here show full anti-proliferative activity and induction of apoptosis in a medium with 10 % serum. Consequently, we did not perform serum starvation to be able to analyze the mode of action under standard treatment conditions. BT474 cells were seeded 24 h prior to treatment. Cells were treated for 2 h with 100 nM of the indicated $Alexa_{488}$-conjgated DARPins at 37 °C to allow for internalization. Afterwards, cells were washed with PBS and harvested by Accutase treatment, washed with medium and washed 2 times with PBS. Afterwards, 25 µg/ml anti-$Alexa_{488}$ quenching antibody was added to the cells and samples were incubated for 30 min at 4 °C. Thereafter, the amount of internalized DARPins was measured by FACS. Additionally, to perform the ErbB2 receptor pulse-chase assay, cells were resuspended in warm medium containing 25 µg/ml anti-$Alexa_{488}$ quenching antibody and incubated at 37 °C for the indicated times. Afterwards, cells were returned to ice and washed 2 times with cold PBS and the remaining fluorescence signals (internalized DARPins) were detected by FACS.

Cell Migration Assay

SKBR3 cell motility was assessed using a wound healing assay. Cells were seeded in 35 mm dishes at $2x10^5$ cells/cm^2 and allowed to adhere for 8 h. An open "scratch" of 500 µm was applied using sterile pipette tips. Dislodged cells were removed by 3 washes with complete culture medium, and cells were incubated for 2 h with 100 nM targeting agents prior to addition of 1 nM HRG or 4 nM EGF. Migration into the open area was documented at 48 h post-scratching. The open scratched area of 8 wound fields per treatment was quantified with TScratch software[13].

Invasion Assay

Invasion assays were performed using BD Biocoat 24-well invasion chambers containing 8 µm pore PET membranes coated with basement membrane matrix (BD Matrigel), following the manufacturer's instructions. Briefly, 1 x 10^5 SKBR3 cells in serum-free medium were introduced into the upper compartment and co-incubated with mixtures of ErbB2-targeting agents and HRG. The lower compartment was filled with medium supplemented with 20% FCS, serving as chemoattractant. After 72 h of incubation, the non-invading cells were wiped off with a cotton swab from the upper membrane surface. The invading cells attached to the lower surface were fixed with 4% paraformaldehyde, stained with 0.5% crystal violet, and counted in ten randomly selected microscopic fields.

Modified XTT Cell Proliferation Assay

The XTT reagent was prepared according to the manufacturer's protocol (Roche). Medium of the 3D-cultured BT474 cells, embedded in BD Matrigel, was removed and 1 ml of XTT reagent was added to each well of the 12-well plate. The XTT reagent was developed for more than 4 h until red staining in the mock treatment became visible and afterwards 800 ul of the developed XTT solution was transferred into a fresh 12-well plate, without disturbing the Matrigel. The 12-well plate was measured in a microplate reader with a respective plate setup.

Caspase Activity

BT474 cells were seeded at 10,000 cells/cm^2 16 h before treatment in RPMI1640 medium containing 10 % FCS. Cells were treated with anti-ErbB2 agents for the indicated times and washed 2 times with ice-cold PBS. Cells were scraped in PBS, centrifuged at 300 rpm and lysed in PBS containing 1% Triton X-100 for 10 min at 4°C. Afterwards, lysates were centrifuged at 20,000 *g* at 4 °C for 10 min and subsequently frozen in liquid nitrogen. Protein concentrations were determined by BCA assays (Pierce). Caspase 3/7, 8 and 9 luminescence substrates (Promega) were prepared according to the manufacturer's protocol. 50 µl of luminescence substrates and the supplied inhibitors were mixed with 50 µl of 0.1 mg/ml lysates in a chilled white 96-well plate (Nunc) and fluorescence was detected on an ELISA plate reader (Tecan, Infinite M1000).

Transfection of siRNA: ErbB3, Grb2 and RAS Knock-Down

Knockdown of ErbB3, Grb2 and H/K-RAS expression was performed by transfection of breast cancer cells using TransIT-siQUEST (Mirus) according to the manufacturer’s protocol. Briefly, for a final concentration of 50 nM of the individual siRNA’s in either 10 ml (10 mm dish) or in 30 ml (150 mm dish), recommended amount of siQUEST transfection reagent and medium (RPMI-1640 w.o. additives) was premixed and siRNA was added for complex formation according to the manufacturer's recommendations for 15 min. Afterwards, BT474, AU565, HCC1419 and SKBR3 cells, which were seeded 24 h prior treatment, were transfected for 1 h in a cell incubator in full medium and subsequently cells were treated with the indicated agents. Treatments were applied in the following order: first addition of siRNA transfection mix (or siRNA cocktail), second addition of small molecule inhibitors and third addition of anti-ErbbB2 binding agents. Double knockdowns were performed simultaneously.

Active RAS Detection Assay and p110α-RAS Co-immunoprecipitation

RAS activation assays were performed using the Active-GTPase Detection kit (Cell Signaling). Briefly, cell lysates were immunoprecipitated with a GST fusion protein corresponding to the

RAS binding domain of Raf-1 bound to glutathione-agarose to identify RAS-GTP. Addition of GTPγS and GDP to cell lysates prior to immunoprecipitation served as positive and negative controls, respectively. The complex p110-RAS co-immunoprecipitation was performed analogously to Active-GTPase detection. Using anti-p110α antibody instead of GST-Raf1-RBD and protein-A agarose (BioRad) instead of glutathione resin. For both assays, IP was performed for 1 h at 4°C and afterwards agarose was eluted with reducing SDS sample buffer and subjected to SDS-PAGE and western blot. The pan-RAS and p110α protein was detected by respective primary antibodies (Cell Signaling) and secondary anti-mouse (Pierce) and anti-rabbit (Sigma) HRP conjugated antibody using a FujiFilm Las-3000.

Doxycycline inducible expression of dominant active and dominant negative Src

DNA strings with N-terminally HA-tagged human dominant active Src (Y527F) or N-terminally HA-tagged human dominant negative Src (Y527F/K295M), were designed according to previously published results from human full length Src[14] and purchased from Integrated DNA Technologies (IDT). Strings of HA-tagged Src including a 5’ Kozak consensus sequence were cloned via HindIII/BamH1 into multiple cloning site of doxycycline inducible vector and confirmed by sequencing. BT474 cells were first established with pcDNA6/TR and afterwards transfected with pcDNA4/TO Src (dom. act.; dom. neg.) and single clones were selected for several week according to manufacture protocol 2011-11-08 (Invitrogen, Life Technology) using manufactures antibiotics blasticidin or zeocine, respectively. Medium was supplemented afterwards with 2.5 µg/ml blasticidin and 10 µg/ml zeocine to maintain expression. Expression was induced by addition of doxycycline (Sigma Aldrich) 24 h before treatment at a concentration of 1 µg/ml.

Mouse Housing

8-week-old female SCID beige mice were obtained from Charles River Laboratories. The mice were free of all viral, bacterial and parasitic pathogens listed in the Federation of European Laboratory Animal Associations (FELASA) recommendations. Animals were kept in type III plastic cages (425 x 266 x 150 mm, floor area 820 cm²) with autoclaved dust-free wooden bedding (80-90 g/cage) (Schill AG, Muttenz, Switzerland) and autoclaved paper tissues

(2/cage) and a paper house as nesting material. They were fed a pelleted mouse diet (Kliba No. 3431, Provimi Kliba, Kaiseraugst, Switzerland) *ad libitum* and had unrestricted access to sterilized drinking water. The light/dark cycle in the room consisted of 12/12 h with artificial light (40 Lux in the cage) from 07:00 to 19:00 h. The temperature was 21±1°C, with a relative humidity of 50±5% and with 15 complete changes of filtered air per hour (HEPA H 14 filter, Vokes-Air, Uster, Switzerland); the air pressure was controlled at 50 Pa. The studies were approved by the Cantonal Veterinary Office (Zurich, Switzerland). Housing and experimental procedures were in accordance with the Swiss animal protection law and conformed to the European Convention for the protection of vertebrate animals used for experimental and other scientific purposes (Council of Europe no. 123 Strasbourg 1985). The animals had a two weeks adaptation time after the arrival at the animal facility.

Mouse Perfusion

After anesthesia by intraperitoneal injection of Ketasol (100 mg/kg body weight) / Xylasol (10 mg/kg body weight) mice were perfused with paraformaldehyde. The abdominal and thoracal cavity was opened, followed by incision of the left ventricle. NaCl (0.9%) was perfused (gravity flow – height 70 cm); by simultaneously opening of the right atrium, perfusion was performed until the blood became colorless (approx. 1-3 min). Perfusion was continued with 4 % paraformaldehyde solution for 5 min. Tumors were dissected and additionally fixed for 2-3 h at RT with 4% PFA.

Histology and Immunohistochemistry

Five-µm sections from paraformaldehyde-fixed and paraffin embedded (using the facility of the Institute of Anatomy, University of Zurich, with standard histology protocols) BT474 tumors were performed and stained with a Hematoxylin solution according to Gill II and Eosin Y solution (0.5 %) in water (Carl Roth) using routine methods. To stain with antibodies specific for ErbB2 (HER2 monoclonal mouse antibody) (Millipore), cleaved caspase-3 (polyclonal rabbit antibody) (Cell Signaling), phospho-ErbB2 (Tyr1221/1222) (rabbit monoclonal antibody) (Cell Signaling), phospho-p44/42 MAPK (Erk1/2) (Thyr202/204) (rabbit monoclonal antibody) (Cell Signaling), phospho-AKT (Ser473) (rabbit monoclonal antibody)

(Cell Signaling) and TUNEL reagent (In Situ Cell Death Detection Kit, Fluorescein, Roche) slides were dried overnight at 37°C and baked in an oven at 60°C for 1 h. After deparaffinization and rehydration (xylene and alcohol bath solutions) the tissue sections were exposed to heat-induced antigen retrieval by incubating slides in a microwave oven (5 min 350 W, 5 min 800 W).

The DAKO EnVision TM+ System Peroxidase (DAB) Kit (K4011) was used for cleaved caspase-3, phospho-p44/42 MAPK and phospho-AKT staining. Endogenous peroxidase activity was blocked by treating the sections with 0.3% (v/v) hydrogen peroxide in buffer for 10 min. The primary antibody was diluted, as recommended by the manufacturer, 1:100 in Tris-buffered saline (TBS), 50 mM, pH 7.5 with 1% bovine serum albumin and the tissue sections were incubated with antibody for 1 hour at room temperature. TBS with 0.1 % Tween-20 was used for the washing steps. The subsequent steps of the staining procedure followed the standard protocol of the kit. After specific staining, all slides were counterstained with Hematoxylin (according to Gill II), mounted and cover-slipped with Histomount mounting medium (Invitrogen Life Technologies)

The In Situ Cell Death Detection Kit was used according to the protocol provided by the manufacturer (Roche). For ErbB2 and phospho-ErbB2 staining, the primary antibody was detected with Alexa488-conjugated goat anti-mouse antibody or goat anti rabbit Oregon Green antibody (Invitrogen), respectively, and nuclei were stained with 4′,6-diamidino-2-phenylindole (DAPI) using standard IHC protocols.

Serum Half-life Quantification of PEGylated DARPins

Quantification of bispecific PEGylated DARPins in the serum of SCID-beige mice was performed by sandwich ELISA. PEGylated DARPins were injected intravenously in the tail vein of the mice at a concentration of 20, 10 and 5 mg/kg. After 1, 4, 24, 48 and 72 h, small blood samples were taken from tail tip according to the Federation of European Laboratory Animal Associations (FELASA) recommendations. Serum was cleared by centrifugation for 10 min at 3000 *g* and samples were snap-frozen and stored at -80°C. ELISA was performed by coating anti-RGSHis$_6$ antibody (QIAGEN) 1:500 on a MaxiSorp plate (Nunc), the plate was washed 3 times with PBS containing 0.1 % Tween and mouse serum was applied for 1 h at RT. Afterwards, the plate was washed 3 times with PBS containing 0.1 % Tween, primary anti-DARPin rabbit serum was added 1:5000 and incubated for 1 h at RT. The plate was

washed 3 times with PBS containing 0.1 % Tween. Goat anti-rabbit secondary antibody labeled with HRP (Sigma Aldrich) was applied 1:10000 and incubated for 1 h at RT. The plate was washed 3 times with PBS containing 0.1 % Tween. Amplex Red substrate (Invitrogen) was added according to the manufacturer's protocol. Fluorescence was detected on a TECAN Infinite M1000 Pro instrument. The serum concentration of PEGylated DARPins was determined by a reference titration of the same PEGylated DARPin.

Statistical Analysis and Software

Unless stated else in the figure caption, error bars represent mean ± standard deviation. Statistical analyses were performed in Sigma Plot 12.5 (Systat). Student’s t-test (two tailed) was applied for comparisons of two groups that both followed the normal distribution with equal variances. Welch’s t-test (two tailed) was applied for comparison of two groups that both followed the normal distribution with unequal variances. Mann-Whitney rank sum test was performed for comparison of two groups that did not follow the normal distribution. ANOVA and post-hoc Dunnett’s test was performed for multiple comparisons versus a control group. In addition, GraphPad Prism 6 (GraphPad) and Origin 9 (Origin Lab) were used to create plots. FlowJo v7.2.5 was used for cell cycle analysis. ImageJ v1.47 (NIH), Cell Profiler 2.1.0 (Broad Institute), NIS Elements (Nikon) and Imaris 7.5 (Oxford Instruments) were used for picture analysis.

Supplementary References

1 Eigenbrot, C., Ultsch, M., Dubnovitsky, A., Abrahmsen, L. & Hard, T. Structural basis for high-affinity HER2 receptor binding by an engineered protein. *P Natl Acad Sci USA* **107**, 15039-15044, doi:10.1073/pnas.1005025107 (2010).

2 Boersma, Y. L., Chao, G., Steiner, D., Wittrup, K. D. & Plückthun, A. Bispecific designed ankyrin repeat proteins (DARPins) targeting epidermal growth factor receptor inhibit A431 cell proliferation and receptor recycling. *The Journal of biological chemistry* **286**, 41273-41285, doi:10.1074/jbc.M111.293266 (2011).

3 Hackel, B. J., Neil, J. R., White, F. M. & Wittrup, K. D. Epidermal growth factor receptor downregulation by small heterodimeric binding proteins. *Protein Eng Des Sel* **25**, 47-57, doi:10.1093/protein/gzr056 (2012).

4 Zahnd, C. *et al.* Efficient tumor targeting with high-affinity designed ankyrin repeat proteins: effects of affinity and molecular size. *Cancer Res* **70**, 1595-1605, doi:10.1158/0008-5472.CAN-09-2724 (2010).

5 Binz, H. K. *et al.* High-affinity binders selected from designed ankyrin repeat protein libraries. *Nat Biotechnol* **22**, 575-582, doi:10.1038/nbt962 (2004).

6 Forrer, P., Stumpp, M. T., Binz, H. K. & Pluckthun, A. A novel strategy to design binding molecules harnessing the modular nature of repeat proteins. *FEBS Lett* **539**, 2-6 (2003).

7 Steiner, D., Forrer, P. & Plückthun, A. Efficient selection of DARPins with sub-nanomolar affinities using SRP phage display. *J Mol Biol* **382**, 1211-1227, doi:10.1016/j.jmb.2008.07.085 (2008).

8 Jost, C. *et al.* Structural basis for eliciting a cytotoxic effect in HER2-overexpressing cancer cells via binding to the extracellular domain of HER2. *Structure* **21**, 1979-1991, doi:10.1016/j.str.2013.08.020 (2013).

9 Zahnd, C. *et al.* A designed ankyrin repeat protein evolved to picomolar affinity to Her2. *J Mol Biol* **369**, 1015-1028, doi:10.1016/j.jmb.2007.03.028 (2007).

10 Zahnd, C., Pecorari, F., Straumann, N., Wyler, E. & Plückthun, A. Selection and characterization of Her2 binding-designed ankyrin repeat proteins. *J Biol Chem* **281**, 35167-35175, doi:10.1074/jbc.M602547200 (2006).

11 Tamaskovic, R., Simon, M., Stefan, N., Schwill, M. & Plückthun, A. Designed ankyrin repeat proteins (DARPins) from research to therapy. *Methods Enzymol* **503**, 101-134, doi:10.1016/B978-0-12-396962-0.00005-7 (2012).

12 Spangler, J. B. *et al.* Combination antibody treatment down-regulates epidermal growth factor receptor by inhibiting endosomal recycling. *P Natl Acad Sci USA* **107**, 13252-13257, doi:10.1073/pnas.0913476107 (2010).

13 Gebäck, T., Schulz, M. M., Koumoutsakos, P. & Detmar, M. TScratch: a novel and simple software tool for automated analysis of monolayer wound healing assays. *BioTechniques* **46**, 265-274, doi:10.2144/000113083 (2009).

14 Gonzalez, L. *et al.* Role of c-Src in human MCF7 breast cancer cell tumorigenesis. *J Biol Chem* **281**, 20851-20864, doi:10.1074/jbc.M601570200 (2006).
